# The genome of newly classified *Ochroconis mirabilis*: Insights into fungal adaptation to different living conditions

**DOI:** 10.1186/s12864-016-2409-8

**Published:** 2016-02-03

**Authors:** Su Mei Yew, Chai Ling Chan, Chee Sian Kuan, Yue Fen Toh, Yun Fong Ngeow, Shiang Ling Na, Kok Wei Lee, Chee-Choong Hoh, Wai-Yan Yee, Kee Peng Ng

**Affiliations:** Department of Medical Microbiology, Faculty of Medicine, University of Malaya, 50603 Kuala Lumpur, Malaysia; Codon Genomics SB, No. 26, Jalan Dutamas 7, Taman Dutamas, Balakong, 43200 Seri Kembangan, Selangor Darul Ehsan Malaysia; Department of Pre-Clinical Sciences, Faculty of Medicine and Health Sciences, Universiti Tunku Abdul Rahman, Bandar Sungai Long, 43000 Kajang, Selangor Darul Ehsan Malaysia

**Keywords:** Dematiaceous, *Ochroconis mirabilis*, Genome sequence

## Abstract

**Background:**

*Ochroconis mirabilis*, a recently introduced water-borne dematiaceous fungus, is occasionally isolated from human skin lesions and nails. We identified an isolate of *O. mirabilis* from a skin scraping with morphological and molecular studies. Its genome was then sequenced and analysed for genetic features related to classification and biological characteristics.

**Results:**

UM 578 was identified as *O. mirabilis* based on morphology and internal transcribed spacer (ITS)-based phylogeny. The 34.61 Mb assembled genome with 13,435 predicted genes showed less efficiency of this isolate in plant cell wall degradation. Results from the peptidase comparison analysis with reported keratin-degrading peptidases from dermatophytes suggest that UM 578 is very unlikely to be utilising these peptidases to survive in the host. Nevertheless, we have identified peptidases from M10A, M12A and S33 families that may allow UM 578 to invade its host via extracellular matrix and collagen degradation. Furthermore, the lipases in UM 578 may have a role in supporting the fungus in host invasion. This fungus has the potential ability to synthesise melanin via the 1,8-dihydroxynaphthalene (DHN)-melanin pathway and to produce mycotoxins. The mating ability of this fungus was also inspected in this study and a mating type gene containing alpha domain was identified. This fungus is likely to produce taurine that is required in osmoregulation. The expanded gene family encoding the taurine catabolism dioxygenase TauD/TdfA domain suggests the utilisation of taurine under sulfate starvation. The expanded glutathione-S-transferase domains and RTA1-like protein families indicate the selection of genes in UM 578 towards adaptation in hostile environments.

**Conclusions:**

The genomic analysis of *O. mirabilis* UM 578 provides a better understanding of fungal survival tactics in different habitats.

**Electronic supplementary material:**

The online version of this article (doi:10.1186/s12864-016-2409-8) contains supplementary material, which is available to authorized users.

## Background

The genus *Ochroconis* comprises oligotrophic species found in litter, soil and moist surfaces that have also been associated with occasional opportunistic infections in humans and animals [1]. Macroscopically, these fungi are characterised by red-brown exudates in the culture medium. Under microscopic examination, rhexolytically liberated conidia with open denticles and frills remaining on the conidial bases are commonly observed [2]. Their taxonomic classification has been problematic. The fungi have been suggested to be grouped within Chaetothyriales [3] or as unclassified anamorphic ascomycetes [4]. Machouart et al. [1], however, used multigene (mtSSU, nuLSU, nuSSU, RPB2 region 5–7 and RPB2 region 7–11) phylogenetic analysis to classify the fungi in the family Sympoventuriaceae of class Dothideomycetes. From the original four species, *O. gallopava*, *O. constricta*, *O. humicola* and *O. tshawytschae* [2], the genus has expanded to include at least 13 species [5]. Following intensive revision based on molecular, morphological and ecological comparisons, a new genus *Verruconis* was proposed for the thermophilic oligotrophs *O. gallopava*, *O. verruculosa* and *O. calidifluminalis* [5]. The remaining *Ochroconis* species are mesophilic saprobes, causing infections mainly in cold-blooded animals such as fish and frogs, but also occasionally in warm-blooded vertebrates including humans [5]. Two of the species, *O. lascauxensis* and *O. anomala* had been isolated from Lascaux Cave, causing black stains on cave sediments, walls and paintings [6]. A strain of *O. constricta* from soil was reported to have keratinolytic activity [7].

*O. mirabilis* has been previously identified as *O. constricta*, a species that has been isolated mostly from water (aquatic vertebrates, sea sponge and sea fan), domestic environments (bathrooms and balconies), and human skin and nails [4, 5]. We previously isolated five strains of *O. constricta* (UM 314, UM 324, UM 326, UM 329 and UM 578) [8, 9]. The genome of UM 578 was sequenced, and preliminary observations on its genome were reported [9]. In this study, UM 578 was re-identified as *O. mirabilis* based on phylogenetic and phylogenomic analyses. Moreover, we analysed its genome content in-depth. Additional studies on carbohydrate enzymes, lipases, secondary metabolite backbone genes, mating type genes, comparative gene families and protein families expansion and contraction are presented in this study. These genomic features are possibly associated with the ability of the species to thrive in different environments.

## Results and discussion

### Morphological and molecular identification

**Fig. 1:**
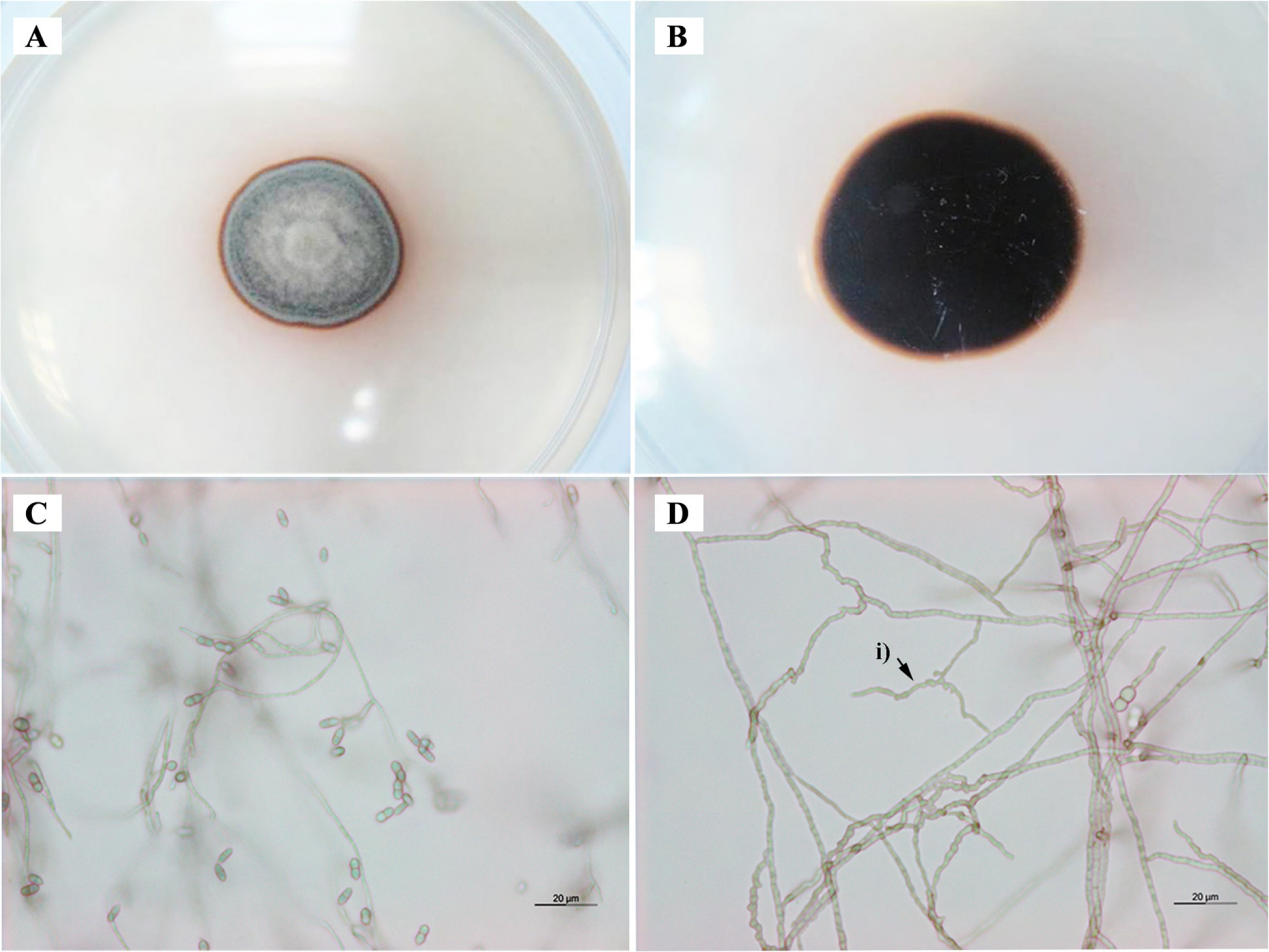
Macroscopic and microscopic characteristics of UM 578. Culture characteristics of UM 578 (**a**) from the front and (**b**) from the reverse on SDA. Microscopic characteristics of the (**c**) conidia and (**d**) i) anastomosing hyphae. Bars 20 μm

Microscopic examination revealed rhexolytic liberation of conidia, unbranched, cylindrical to acicular conidiophores; smooth-walled to verruculose, subhyaline to pale brown coloured conidia that were constricted at the septum; and the presence of anastomosing hyphae as described by Samerpitak et al. [5] (Fig. 1). The colony of UM 578 was grey brown and raised in the middle with red-brown colour in the circumference and the medium surrounding the colony. The reverse side of the colony was dark brown in colour and did not grow into the agar.

**Fig. 2:**
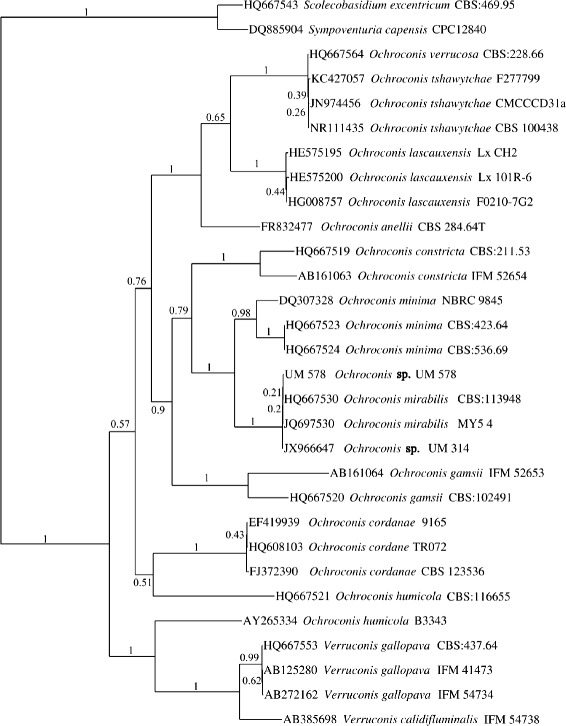
ITS-based phylogenetic tree of species from the genus *Ochroconis. Ochroconis* spp. ITS-based phylogenetic tree generated by Bayesian analysis based on 100 sampling frequency for a total of 500,000 generations. *Scolecobasidium excentricum* and *Sympoventuria capensis* were used as outgroup strains. UM 578 was resolved as *O. mirabilis*. The accession number for UM 578 ITS sequence is KF639587

UM 578 was previously identified as *O. constricta*. Here, we re-examined its identity using an ITS-based phylogenetic tree. Phylogenetic analysis showed UM 578 clustering with UM 314 within the *O. mirabilis* cluster (Fig. 2).

**Fig. 3:**
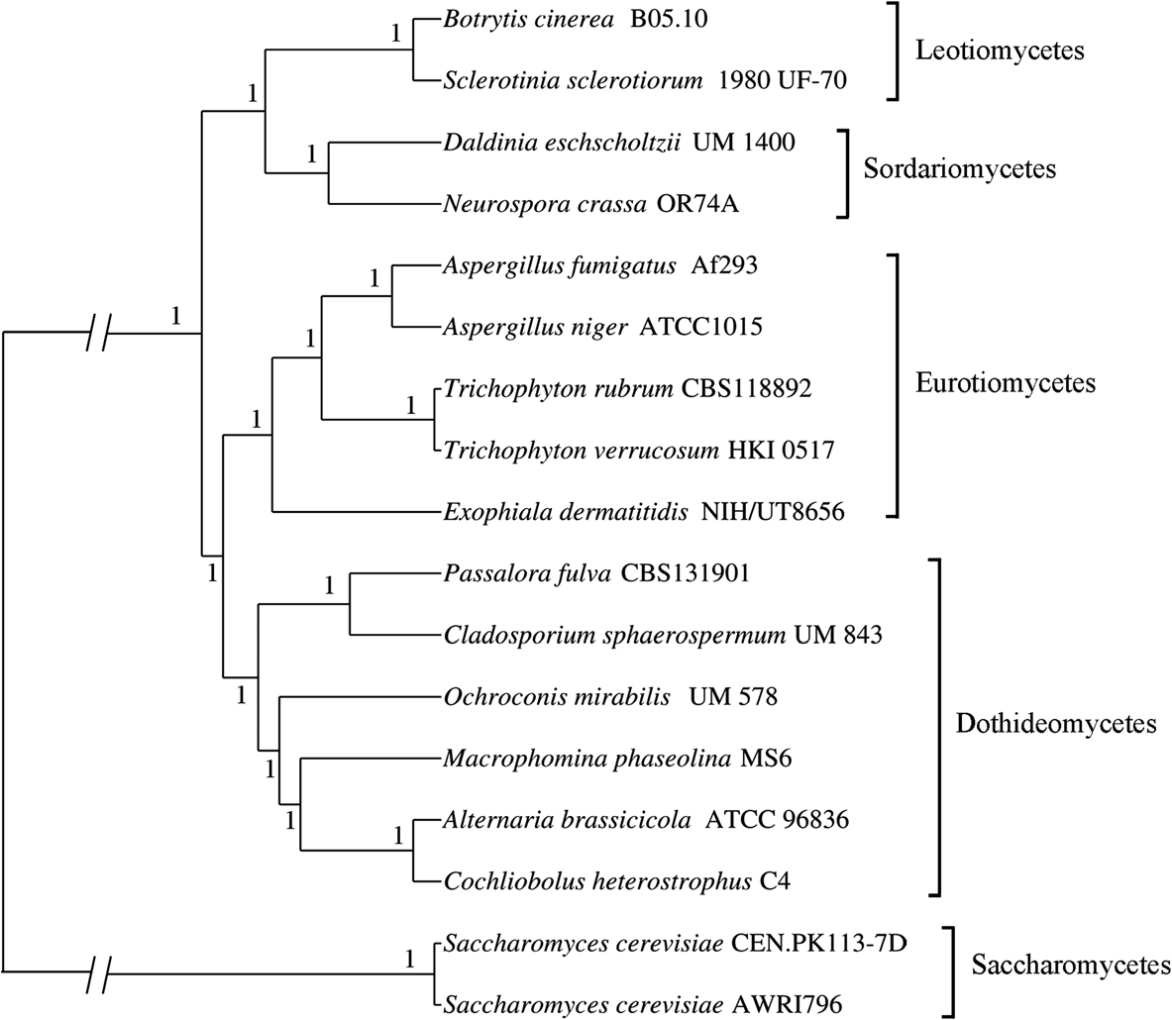
Phylogenomic tree of UM 578 and other fungi. The phylogenomic tree was constructed using 16 publicly available fungal genomes from class Eurotiomycetes, Sordariomycetes and Dothidemycetes with two fungal genomes from class Saccharomycetes as outgroup. The tree was generated using Bayesian analysis. UM 578 was found clustered within class Dothideomycetes

We constructed a phylogenomic tree with protein sequences from 17 fungal genomes from different classes, including two Leotiomycetes, two Sodariomycetes, five Eurotiomycetes, six Dothideomycetes and two Saccharomycetes as outgroups (Fig. 3; Additional file 1: Table S1). A total of 181,384 proteins were clustered into 18,666 orthologous clusters with 917 single-copy orthologues identified. The phylogenomic tree is consistent with that in a previous study [1] which showed *O. mirabilis* UM 578 within class Dothideomycetes.

### Genome assembly, gene models and transposable elements

The 500-bp and 5-kb insert libraries generated 22,277,778 reads and 11,222,224 reads [9], respectively using Illumina HiSeq 2000. The sequencing coverage for the combined sequenced reads was 80-fold (Table 1). The reads were assembled into 603 contigs, with 544 contigs having size ≥200 bp. The contigs were then scaffolded into 163 scaffolds based on the paired-end information from both libraries. The assembly size of UM 578 was ~34.61 Mb, with a scaffold N50 of 1,170,353 bp. The assembled genome had a GC content of 51.84 %. A total of 13,435 genes were predicted using GeneMark-ES version 2.3e [10], with an average gene length of 1411 bp. A total of 14 rRNAs and 71 tRNAs were predicted in the genome.

**Table 1:** Sequencing statistics and genome features of UM 578

	*Ochroconis mirabilis* UM 578
Reads from 500 bp insert library (Mb)	22.28
Reads from 5 kb insert library (Mb)	11.22
Total Reads (Mb)	33.50
Assembly size (Mb)	34.61
Number of contigs (≥200 bp)	544
Contigs size (N50) (bp)	220443
Number of scaffolds (≥1000 bp)	163
Scaffolds size (N50) (bp)	1170353
G + C content (%)	51.84
Number of predicted genes (≥99 bp)	13435
Average gene length (bp)	1411
Average number of exons per gene	2.57
rRNA	14
tRNA	71

There were 285 class I retrotransposons and 10 class II DNA transposons encompassing 1.09 % of the assembled genome (Table 2). Gypsy and Copia from the class I retrotransposons and Tc1-Mariner from the class II DNA transposons are reported to be the most abundant transposable elements in fungal genomes [11]. As seen in Table 2, Gypsy type forms the highest number of transposable elements in UM 578, followed by DDE_1 and Ty1_Copia while helitronORF type forms the largest number in class II transposons. Although the repetitive elements described here may not represent all transposable elements in the genome of this isolate owing to the limitation of Illumina technology [12], they provide an idea of the type of transposable elements identified in this genome. The composition and organisation of repetitive elements in the genome enable the delineation of the best strategy for sequencing the whole genome [13].

**Table 2:** Transposable elements predicted in UM 578

Class	Family name	Total number	Total bases	Percentage (%)
I	DDE_1	71	57925	15.41
	gypsy	166	233984	62.25
	LINE	17	32821	8.73
	TY1_Copia	31	41543	11.05
II	cacta	1	161	0.04
	hAT	1	497	0.13
	helitronORF	5	7321	1.92
	mariner	1	680	0.18
	MuDR_A_B	2	1063	0.28
Total		295	375905	100

### Gene annotation

**Fig. 4:**
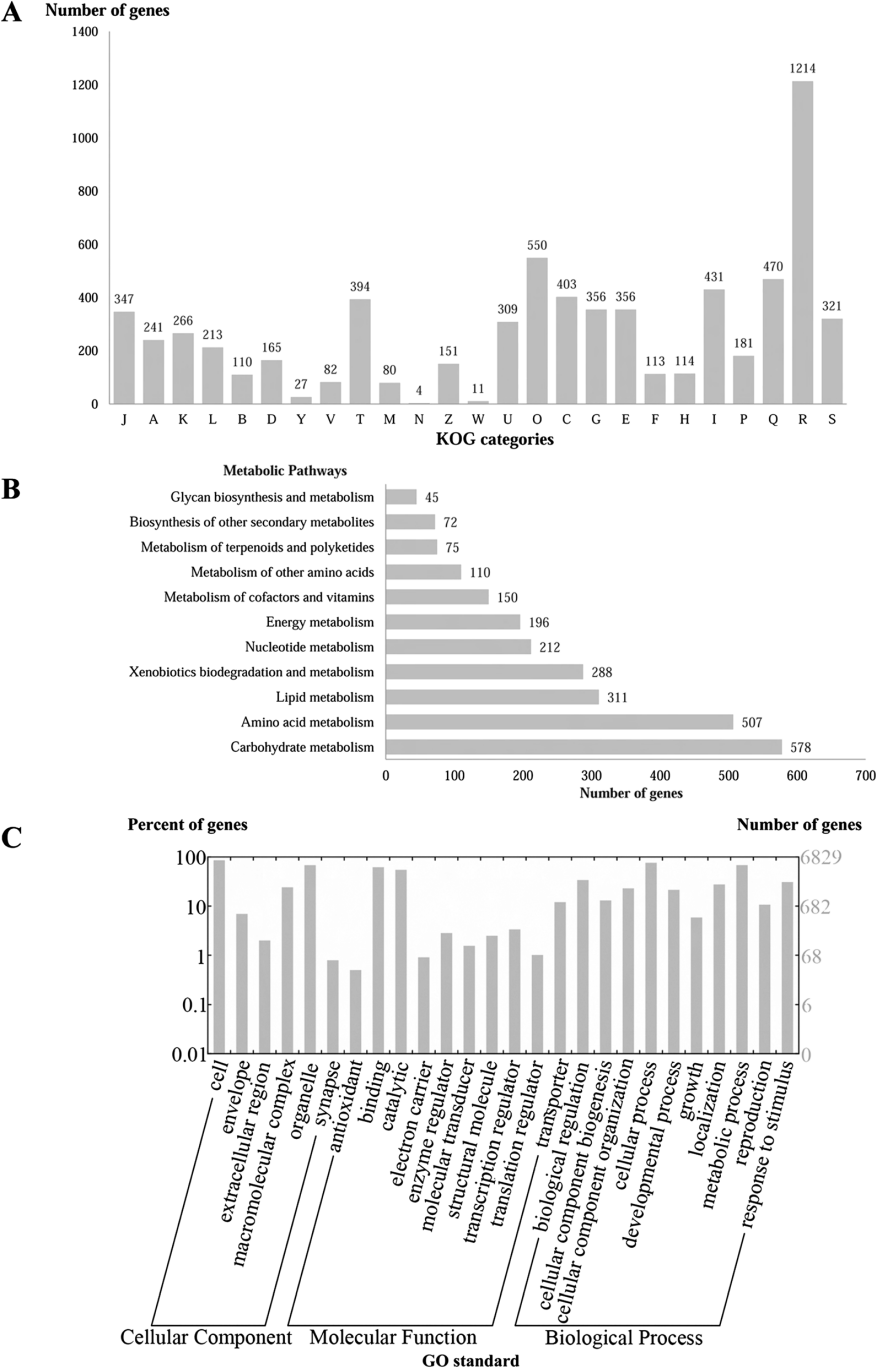
KOG, KEGG and GO classifications of predicted genes in *O. mirabilis* UM 578. **a** Distribution of KOG classes. i) Information storage and processing caterory: J, Translation, ribosomal structure and biogenesis; A, RNA processing and modification; K, Transcription; L, Replication, recombination and repair and B, Chromatin structure and dynamics. ii) Cellular processes and signalling category: D, Cell cycle control, cell division, chromosome partitioning; Y, Nuclear structure; V, Defense mechanisms; T, Signal transduction mechanisms; M, Cell wall/membrane/envelope biogenesis; N, Cell motility; Z, Cytoskeleton; W, Extracellular structures; U, Intracellular trafficking, secretion, and vesicular transport and O, Posttranslational modification, protein turnover, chaperones. iii) Metabolism category: C, Energy production and conversion; G, Carbohydrate transport and metabolism; E, Amino acid transport and metabolism; F, Nucleotide transport and metabolism; H, Coenzyme transport and metabolism; I, Lipid transport and metabolism; P, Inorganic ion transport and metabolism and Q, Secondary metabolites biosynthesis, transport and catabolism. iv) Poorly characterised category: R, General function prediction only and S, Function unknown. **b** KEGG metabolic pathway distribution. **c** Distribution of GO annotations

In the functional categorisation using EuKaryotic Orthologous Group (KOG), 6078 predicted genes were redundantly assigned into KOG classifications (Fig. 4a), of which, 1535 genes were annotated as poorly characterised proteins, 1177 assignments were in the category of Information Storage and Processing, 1173 in the Cellular Processes and Signalling category and 2424 in the Metabolism category. Of the eight KOG classifications in the Metabolism category, 470 genes were annotated to the Secondary Metabolites Biosynthesis, Transport and Catabolism [Q], 431 genes annotated to Lipid Transport and Metabolism [I] and 403 genes annotated to Energy Production and Conversion [C], composing the top three classifications in this category. In class Q, there were 57 genes annotated as flavin-containing monooxygenase. Flavin-containing monooxygenases are widely found in many organisms and have multiple biological functions. These enzymes in UM 578 might play a role in the biodegradation of environmental aromatic compounds, detoxification of drugs and antibiotics, and siderophore biosynthesis [14, 15], processes which provide a survival advantage in the adverse environment.

Of the 1012 predicted genes annotated in the Kyoto Encyclopedia of Genes and Genomes (KEGG) pathway maps, the xenobiotics biodegradation and metabolism is the fourth highest metabolic pathway mapped with predicted genes from this species (288 genes), after carbohydrate metabolism (578 genes), amino acid metabolism (507 genes) and lipid metabolism (311 genes) (Fig. 4b). From the styrene degradation map (Additional file 2: Figure S1), three enzymes were mapped in the degradation of Z-phenylacetaldoxime. The enzymes were nitrilase (EC 3.5.5.1), nitrile hydratase (EC 4.2.1.84) and amidase (EC 3.5.1.4) but no phenylacetaldoxime dehydratase (EC 4.99.1.7). To further inspect the capability of UM 578 in Z-phenylacetaldoxime degradation, a gene annotated as phenylacetaldoxime dehydratase was identified. The gene, UM578_4049 encodes an enzyme belonging to the haem-containing dehydratase family (IPR025702) that has 31 % similarity to the *Bacillus* sp. OxB-1 phenylacetaldoxime dehydratase Oxd [GenBank: P82604] [16] (Additional file 2: Figure S2). Interestingly, a nitrilase (UM578_4050) encoding gene is located adjacent to the *oxd* gene in the genome as observed previously in *Fusarium graminearum* [17], *Bacillus* sp. OxB-1 [16] and *Pseudomonas syringae* [18] (Additional file 2: Figure S3). However, the gene arrangement in UM 578 is different from that in those reported species, and the predicted regulatory protein is missing in UM 578.

Furthermore, we found that UM 578 might be able to degrade cyanamide in the atrazine degradation pathway (Additional file 2: Figure S4). Cyanamide is a reactive substance that seldom occurs in nature and remains undegradable for a long time in an abiotic medium. It is used as a nitrogen fertiliser in the form of calcium salt and hydrogen cyanamide. Cyanamide was also reported to be phytotoxic, bactericidal and fungicidal [19, 20]. In this genome, two genes were identified to encode cyanamide hydratase (UM578_11171) and urease (UM578_4352), respectively and are shown to be expressed (Additional file 2: Figure S5). Cyanamide hydratase catalyses the hydrolysis of cyanamide to urea while urease converts the urea to ammonium. The cyanamide hydratase from the fungus *Myrothecium verrucaria* is well characterised and used in transgenic plants to act as a biocontrol of phytopathogens [20]. Moreover, the end product, urea, is a useful compound that acts as a plant fertiliser to facilitate plant growth [20]. The presence of genes involved in biodegradation shows the adaptation of the fungus towards the occurrence of non-natural compounds in the environment.

In the functional classification of UM 578 genome based on gene ontology (GO), 6829 of 13,435 predicted genes were given a GO assignment. Of these genes, 17,695 were redundantly assigned into Cellular Component Ontology, 14,584 into Molecular Function Ontology and 33,100 into Biological Process Ontology (Fig. 4c). Most of the genes were annotated to Cell (5874 genes) and Organelle (4656 genes) in the Cellular Component Ontology, Binding Activity (4230 genes) and Catalytic Activity (3743 genes) in the Molecular Function Ontology, and Cellular Process (5215 genes) and Metabolic Process (4662 genes) in the Biological Process Ontology. As these are the fundamental components and processes for the viability of an organism, it is not surprising that these genes encompass a large portion of the genome.

### Carbohydrate metabolism

**Fig. 5:**
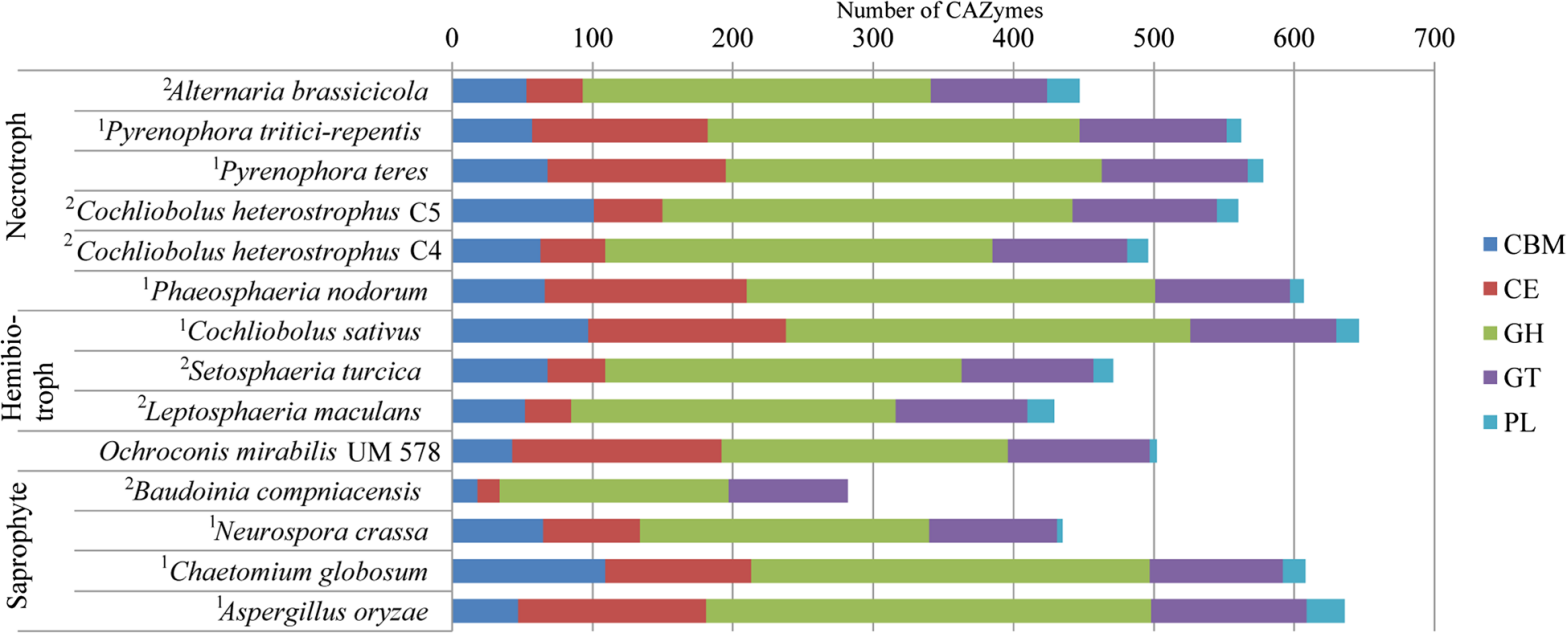
Comparison of CAZyme modules in UM 578 and other fungi with different lifestyles. UM 578 has a higher number of predicted CAZyme modules compared to some of the fungi in different lifestyles. CBM, carbohydrate-binding modules; CE, carbohydrate esterases; GH, glycoside hydrolases; GT, glycosyltransferases; PL, polysaccharide lyases. ^1^Data obtained from Zhao et al. [88]; ^2^Data obtained from Ohm et al. [12]

**Fig. 6:**
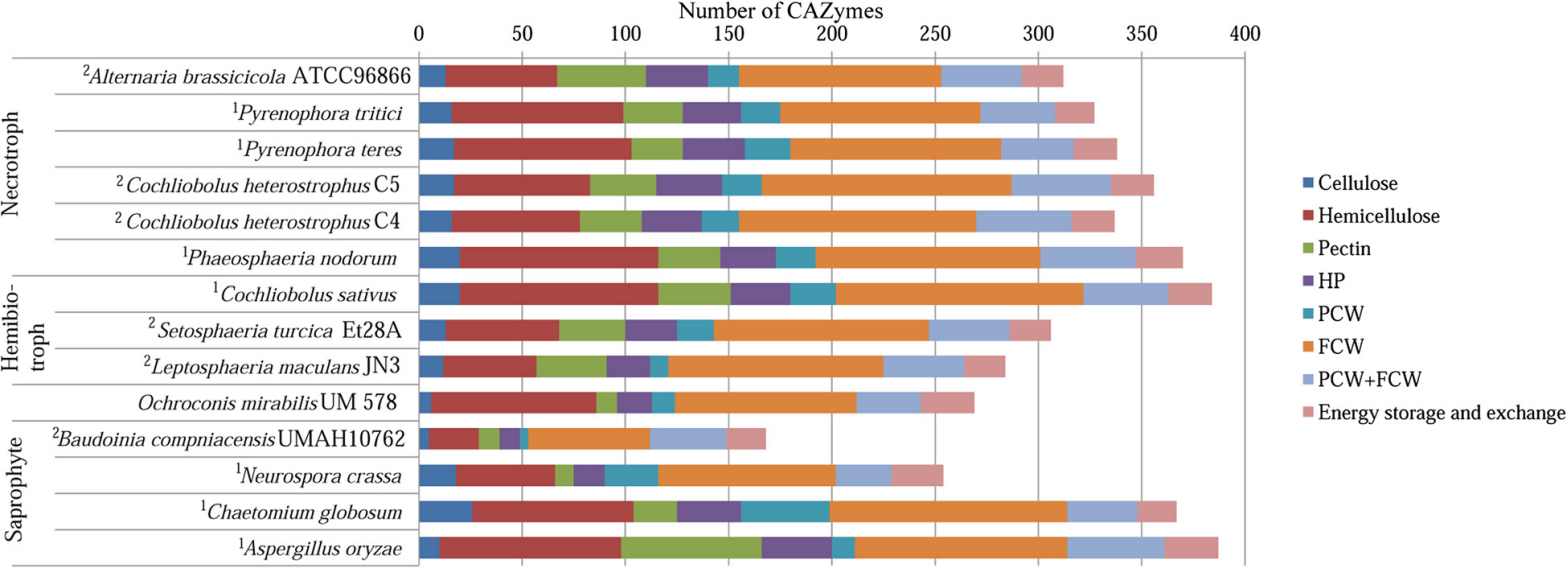
Comparison of CAZyme modules based on substrate specificity in UM 578 and fungi with different lifestyles. UM 578 has a lower number of CAZymes involved in plant cell wall degradation as compared to other fungi. Putative functions of families assigned according to Amselem et al. [21]. HP, Enzymes that degrade hemicellulose or pectin side chain; PCW, Enzymes that involved in plant cell wall degradation and modification; FCW, Enzymes involved in fungal cell wall degradation and modification. ^1^Data obtained from Zhao et al. [88]; ^2^Data obtained from Ohm et al. [12]

Heterotrophic fungi harbour Carbohydrate Active enZymes (CAZymes) to degrade complex carbohydrates from organic matters for nutrient supply. We identified 590 CAZyme catalytic domains in UM 578. The modules comprise 88 domains belonging to auxiliary activities, 43 to carbohydrate-binding modules (CBM), 149 to carbohydrate esterases (CE), 204 to glycoside hydrolases (GH), 101 to glycosyltransferases (GT) and five to polysaccharide lyases (PL) (Additional file 1: Table S2). Based on substrates specificity, UM 578 has a very small number of CAZymes involved in cellulose degradation but a high number of CAZymes involved in hemicellulose degradation (Additional file 1: Table S3), indicating a possible least preference towards cellulosic materials compared to compounds high in hemicellulose. It also harbours a larger number of CAZyme modules as compared to some necrotrophic, hemibiotrophic and saprophytic fungi (Fig. 5). However, it contains less CAZyme modules involved in plant cell wall degradation (Fig. 6). The large number of enzymes involved in hemicellulose degradation present in UM 578 indicates a possible preference of this fungus towards soft plant tissues such as fruits [21]. Some modules such as cellobiohydrolase (GH6 and GH7) involved in cellulose degradation, endo-β-1,4-xylanase (GH11), arabinofuranosidase (GH62) and β-1,4-galactanase (GH53) involved in xylan degradation and, pectin methylesterase (CE8) and rhamnogalacturonan lyase (PL4) involved in pectin degradation were absent in this isolate. This might lead to least efficiency in the degradation of plant cell walls. Among the 31 isolates identified as *O. mirabilis* in the study by Samerpitak et al. [5], only a few were isolated from plants.

### Peptidases

Using MEROPS analysis, we identified 186 peptidases in UM 578, of which, 47 were secreted enzymes. The highest numbers were from the metallopeptidase family (70 peptidases), and the serine peptidase family (56 peptidases) (Additional file 1: Table S4).

As *O. mirabilis* are isolated from human skin lesions and nails [5], we looked for genes encoding keratin degradation proteases and identified seven that are secreted proteases similar to the keratin-associated degradation proteases of the dermatophyte *Trichophyton rubrum*. These peptidases belong to the families M14A, S10, M28 and S9. One gene (UM578_1644) encoding a secreted metallocarboxypeptidase from the family M14A, shows 42 and 41 % identity to M14A peptidase of *T. rubrum* [GenBank: ABW79919] [22] and *Metarhizium anisopliae* [GenBank: AAB68600] [23], respectively. This peptidase contains conserved zinc-binding, substrate binding, catalytic sites and cysteine residues (Additional file 2: Figure S5). A total of six secreted carboxypeptidase S10 family were predicted. Of the putative S10 peptidases, UM578_13449 has the best match to the characterised *T. rubrum* carboxypeptidase SCPA [GenBank: AAS76667] [22] (43 % identity). Multiple sequence alignment showed the conserved active sites in UM 578_13449 (Ser228, Asp439 and His497) with *T. rubrum* SCPA and *Aspergillus fumigatus* Cp1 [GenBank: AAR91697], ortholog of SCPA (Additional file 2: Figure S6). In addition, another S10 peptidase, UM578_7889 was identified similar to *T. rubrum* carboxypeptidase Y (SCPC) [GenBank: AAS76668] [22] with 66 % identity.

Furthermore, two genes encoding leucine aminopeptidase (LAP) from the M28 family, UM578_5513 and UM578_7056 exhibited 51 and 54 % identity to *T. rubrum* LAP1 [GenBank: AAS76670] [24] and LAP2 [GenBank: AAS76669] [24], respectively. Multiple sequence alignment was conducted with LAP1 and LAP2 from *T. rubrum* and *A. fumigatus* that have been reported to have the same hydrolytic activity [24] (Additional file 2: Figures S7 and S8). Both genes have the same catalytic sites as reported. The consensus binding sites for the first and second Zn^2+^ ion in UM578_5513 are His252 and Asp326 and, Glu297 and His424 respectively. Asp264 is the predicted residue bridging the two Zn^2+^ ions. The UM578_7056 first Zn^2+^ ion binding site was predicted at His180 and Asp265, and the second Zn^2+^ ion binding site was predicted at Glu238 and His347. The bridging residue is at Asp199. Lastly, we also identified two genes encoding putative dipeptidyl peptidase IV (DPPIV) and DPPV from family S9. UM578_9285 shares 50 and 53 % identity to DPPIV secreted by *T. rubrum* [GenBank: AAS76665] [24] and *A. fumigatus* [GenBank: AAC34310] [25], respectively. Another gene, UM578_9264 shares 47 and 52 % identity to DPPV of the *T. rubrum* [GenBank: AAN03632] [24] and *A. fumigatus* [GenBank: AAB67282] [26], respectively. Both genes have the same conserved catalytic sites as previously reported [26] (Additional file 2: Figures S9 and S10). The two predicted peptidases have the catalytic triad Ser 619, Asp696, His731 and Ser 566, Asp647, His679.

In addition, we identified a gene (UM578_9214) encoding a putative sulphite efflux pump, *Ssu1* (53.17 % identical to *Arthroderma otae* Ssu1 [GenBank: C5G0E3]). SSU1 is involved in the excretion of sulphite to digest keratin by reducing the disulphide bridges in the cornified cell layers [27]. UM578_9214 has ten membrane-spanning helixes and hydrophilic N- and C- termini, which is consistent with the previous report by Léchenne et al. [27] (Additional file 2: Figure S11).

It has been hypothesised that the presence of multiple endoproteases of subtilisins (S8 family) and fungalysins (M36 family) enable dermatophytes to invade hosts as they share similar sets of peptidases with non-dermatophytes [28–31]. We compared the abundance of peptidases found in keratinophilic dermatophytes and in UM 578 (Additional file 1: Table S5). However, we could not find any similar abundance pattern of peptidase families between UM 578 and the dermatophytes. From the gene families analysis conducted with 17 fungal genomes (Additional file 1: Table S6), we identified shared genes among *T. rubrum*, *T. verrucosum* and UM 578, that are not involved in keratin degradation. Most of the shared genes encode hypothetical proteins while some genes encode the ATPase family associated with various cellular activities, methyltransferase, beta-lactamase, alpha/beta hydrolase, glyoxalase-like, NAD-dependent epimerase and DNA binding proteins. Thus, the secreted proteases in UM 578 do not seem to play a role in the survival of *O. mirabilis* in human skin and nails.

Peptidases from families M10A, M12A and S33 were found to be involved in the degradation of collagen and extracellular matrix [32–36]. In the UM 578 genome, we predicted four peptidases belonging to the M10A family (matrix metallopeptidases), nine zinc metallopeptidases from the astacin family (M12A) and 12 prolyl aminopeptidases (S33). Among the four M10A peptidases, three (UM578_7033, UM578_12210 and UM578_12211) are similar to myroilysin that was reported to have elastinolytic activity and synergistic effect in collagen degradation [32]. The matrix metallopeptidase from *Candida albicans* was reported to degrade fibronectin and type I collagen completely as well as partially degrade laminin and type IV collagen [33, 34]. Astacin family peptidases (family M12A) have diverse functions ranging from digestion of food to the processing of extracellular matrix components [35]. Prolyl aminopeptidases from family S33 free the N-terminal residue from a peptide with a preference towards proline, thus, providing an advantage for the fungus to utilise proline-rich substrates such as collagen [36]. As the domestic environment has been reported as a reservoir for fungi causing human infections [37] and the infection was suggested to occur via moisturised human skin after a shower [4], these peptidases are likely to provide the nutrient source for *O. mirabilis* causing skin infections as described for fungi inhabiting moist indoor reservoirs.

### Lipase activity

Lipases belong to many different protein families that do not show sequence similarity but have the same architecture. Some of these enzymes share similar fold and catalytic machinery [38, 39]. The ubiquitous skin-inhabiting fungus, *Malassezia globosa* is unable to synthesise fatty acids but has multiple lipases that enable it to assimilate lipids from the skin of its human host [40]. We compared putative lipases in UM 578 with those in several skin-inhabiting and non-skin inhabiting fungi (Additional file 1: Table S7) and found the percentage of predicted lipase genes in UM 578 (0.32 %) to be comparable to that predicted in *Candida albicans* (0.30 %), an opportunistic skin coloniser (Table 3). The result suggests the possibility of UM 578 employing these lipases for survival on the skin.

**Table 3:** Summary of predicted lipases for each fungus in the analysis

	Total predicted proteins in genome	Number of predicted lipase	Percentage (%)
*Ochroconis mirabilis* UM 578	13435	43	0.32
*Malassezia globosa*	4286	20	0.47
*Candida albicans*	5752	17	0.30
*Trichophyton rubrum*	8706	14	0.16
*Trichophyton verrucossum*	8086	14	0.17
*Cladosporium sphaerospermum*	9652	21	0.22
*Macrophomina phaseolina*	13806	32	0.23

### Secondary metabolites

To the best of our knowledge, there have been no reports on secondary metabolites produced by *Ochroconis*. In UM 578, we identified a total of 14 secondary metabolite backbone genes comprising five polyketide synthases (PKS), one PKS-like, three nonribosomal peptide synthases (NRPS), four NRPS-like and four dimethylallyl tryptophan synthases (DMAT). Polyketides are secondary metabolites that are formed from small carbon precursor acids whose successive condensation is catalysed by PKS [41] and comprise diverse natural products including antibiotics, pigments and mycotoxins.

Melanin plays a significant role in resistance to abiotic stress and pathogenicity in dematiaceous fungi [42]. We found a potential PKS (UM578_2557) responsible for DHN-melanin biosynthesis. The gene is highly similar to the *C. heterostrophus* PKS18 (70.26 %) [GenBank: AAR90272] [43] and the *Bipolaris oryzae* PKS1 (70.11 %) [GenBank: BAD22832] [44]. UM578_2557 contains five conserved domains comprised of ketosynthase (KS), acyltransferase (AT), acyl carrier protein (ACP) and thioesterase (TE) domains in the order KS-AT-ACP-ACP-TE. The arrangement of the domains in the gene is the same as that in the PKS18 and PKS1 genes [43, 44]. Further inspection of the genes near UM578_2557 revealed two genes annotated as transcription factor *Cmr1* (UM578_2558) and tetrahydroxynaphthalene reductase (UM578_2559), located downstream to the PKS gene (Additional file 2: Figure S12). This strengthens our postulation that UM578_2557 encodes a PKS to synthesise DHN-melanin precursor as most of the genes involved in secondary metabolism are found in the clusters. Moreover, the gene organisation and orientation of the UM 578 melanin gene cluster is similar to those in *C. heterostrophus* and *Alternaria brassicicola* [43]. Another two enzymes that are essential in the DHN-melanin synthesis, scytalone dehydratase (UM578_4032) and trihydroxynaphthalene reductase (UM578_5506), were also found in the genome with 70.63 and 47.58 % identity to *Colletotrichum obiculare* scytalone dehydratase [GenBank: Q00455] and trihydroxynaphthalene reductase [GenBank: P87025], respectively.

On the other hand, putative aflatoxin (AF)/sterigmatocystin (ST) biosynthesis genes were found in the UM 578 genome (Additional file 1: Table S8). Production of the toxic and carcinogenic AF which was previously reported to be limited to *Aspergillus* spp. has recently been reported in *Fusarium kyushuense* [45]. ST, the precursor of AF is produced by diverse fungi [46]. Genetic and biochemical studies suggested that the production of AF, ST together with another toxin dothistromin share common biosynthetic pathways [12, 47]. Dosthistromin is a red toxin that was first isolated from *Dothistroma septosporum*. The disease caused by this toxin is known as Dothistroma needle blight with broad spectrum toxicity against bacteria, fungi, plant and animal cells [48]. Few fungi from the class Dothideomycetes are also known to produce this toxin [47]. However, it should be noted that homologues of AF/ST biosynthetic genes are known to be involved in functions other than the production of red toxin. At this stage of knowledge, the exact role of these putative genes remains unknown.

**Fig. 7:**
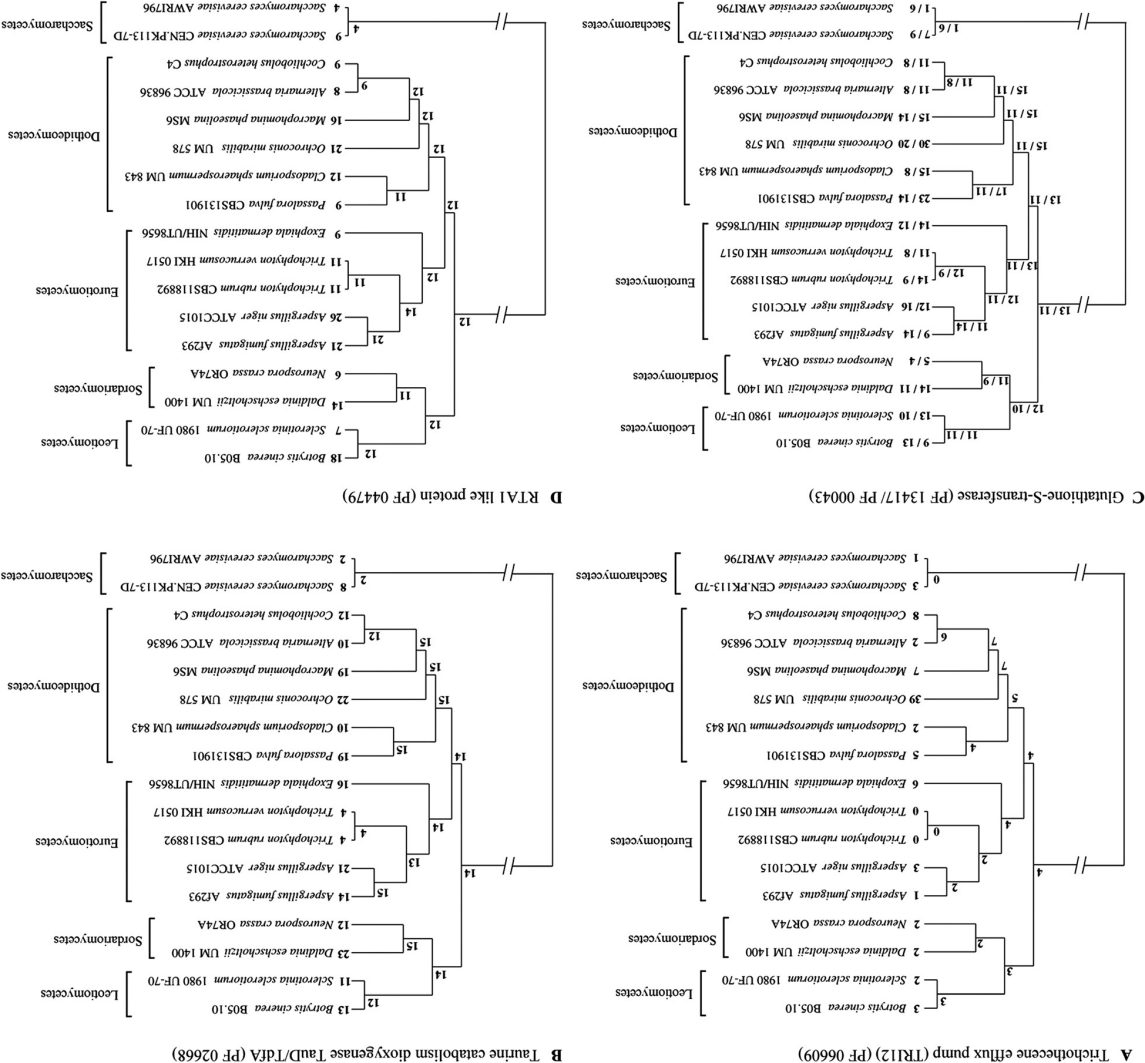
Expansion of selected Pfam families. Predicted proteins were classified into Pfam families and the expansion and contraction of families was analysed using CAFE software. **a** Pfam family of trichothecene efflux pump (TRI12) (PF 06609). **b** Pfam family of taurine catabolism dioxygenase TauD/TfdA (PF 02668). **c** Pfam family of glutathione-S-transferase N-terminal (PF 13417) and C-terminal (PF 00043). **d** Pfam family of RTA1- like protein (PF 04479)

Trichothecenes are a family of mycotoxins consisting of more than 200 structurally related sesquiterpenoid metabolites. The toxins are potent protein synthesis inhibitors and apoptosis inducers in eukaryotic cells. Trichothecenes are usually encountered as contaminants of food and animal feeds. The biosynthesis of trichothecenes involves a series of oxygenation, cyclisation and esterification reactions [49]. Trichothecene-producing fungi are found in the order Hypocreales, including *Stachybotrys*, *Tricothecium*, *Myrothecium*, *Cephalosporium*, *Fusarium* and *Trichoderma*. Species from the genus *Stachybotrys* have been reported as a significant contaminant of the indoor environment and have been associated with damp building-related illness, and the production of satratoxins, roridins and verrucarins types of trichothecenes [50, 51]. The gene families analysis showed a significant number of fungal trichothecene efflux pump (TRI12) domains in the UM 578 genome (Fig. 7a). The function of TRI12 has been postulated to be mainly responsible for self-protection of the fungus by exporting trichothecene outside the cells [52]. We identified the trichodiene synthase encoding gene (UM578_3030) with 48.85 % identity to the *Fusarium asiaticum* trichodiene synthase [GenBank: Q8NIH6]. Other genes encoding enzymes required for trichothecene biosynthesis were located in other regions of the genome (Additional file 1: Table S9). Furthermore, the genes downstream to the putative trichodiene synthase are two proteins containing the cytochrome P450 monooxygenase domain and one protein containing the TRI12 domain (Additional file 2: Figure S13). Based on these results, we hypothesised that the region of genomic DNA that spans 8 kb from UM578_3030 to UM578_3033 is probably a trichothecene biosynthesis cluster for *O. mirabilis*. Overall, these findings show that UM 578 is likely to produce trichothecene.

### Sexual reproduction

Sexual reproduction enables the exchange of genetic material in eukaryotes to produce recombinants that are better adapted to the environment. In the genus *Ochroconis*, the sexual-morph of the species is not well known, and the only species with known teleomorph is *O. sexualis* [5]. In this study, we investigated the involvement of *O. mirabilis* in sexual reproduction by looking for the presence of genes in mating and meiosis. We managed to identify several potential genes participating in the mating process, signalling, fruiting body development and meiosis (Additional file 1: Table S10). An alpha-box domain containing protein (UM578_3656) with 43 % identity to *Fusarium oxysporum MAT-1* gene [GenBank: O59851] [53] involved in activation of alpha-specific genes was identified. UM578_3656 was located adjacent to the genes encoding DNA lyase APN2 (UM578_3657) and cytochrome C oxidase Vla Cox13 (UM578_3658) (Additional file 2: Figure S14). The presence of these two genes in the mating type gene organisation has been reported in *Aspergillus*, *Coccidioides*, *Histoplasma* and dermatophytes [28, 54]. As only *MAT-1* gene was identified in UM 578, this strain could be a heterothallic fungus (Additional file 2: Figure S14).

The homeodomain proteins (HD1 and HD2) and, the alpha-box and HMG domain proteins are the two classes of proteins previously found in the mating type loci and are hypothesised as the ancestral fungal sex determinants. These two classes of sex determinants have undergone gene lost and acquisition in different lineages which resulted in the absence of homeodomain proteins in euascomycetes [54]. A hypothetical protein containing homeodomain was identified upstream of the UM578_3656 gene (Additional file 2: Figure S14). This would be the first euascomycete reported having a putative homeodomain protein in such proximity to the alpha-box. However, further functional validation is required to characterise these proteins. It should also be noted that only pheromone receptors but no pheromone genes were identified in the genome (Additional file 1: Table S10). As pheromone-receptor systems are essential in sexual reproduction [55], there is a possibility that this fungal strain is not able to mate.

### Gene families analysis

The selection of fungal genomes for comparative analysis is based on different lifestyles of the fungal candidates and the characteristics of *O. mirabilis* [4, 5] (Additional file 1: Table S1). A total of 18,666 gene families were identified using OrthoMCL with 1341 families conserved in all 17 fungal genomes. There are 51 families conserved in Dothideomycetes and 286 UM 578 specific gene families in this study set. Among those UM 578 specific gene families, the F-box domain containing genes formed the largest family (14 families), followed by the genes containing heterokaryon incompatibility domain (7 families) (Additional file 1: Table S11).

In the Pfam family expansion and contraction analysis, 61 families were shown to have undergone changes (*P*-value ≤ 0.01 for the whole tree), with 50 families significantly expanded and 11 families contracted in UM 578 (Additional file 1: Table S12). The domains enriched in UM 578 can be functionally categorised into proteins involved in transport functions, protein-protein interactions, transcriptional regulation, oxidoreductase activity and hydrolysis functions. In contrast, the contracted families are domains encoding for PKS backbone enzymes, glycoside hydrolase family 28, and LysM domain (Additional file 1: Table S12). Some of the expanded families are also present in gene families only observed in UM 578 such as taurine catabolism dioxygenase TauD/TdfA, trichothecene efflux pump (TRI12), BTB/POZ domain, antibiotic biosynthesis monooxygenase, and alpha/beta hydrolase fold.

Expansion of genes containing the taurine catabolism dioxygenase TauD/TdfA domain was found with an increase from 15 to 22 copies (Fig. 7b). Four TauD/TdfA genes were found in a UM 578 specific gene family. These genes encode alpha-ketoglutarate-dependent dioxygenase function in the catalysis of taurine to sulfite and aminoacetyldehyde [56]. Taurine is a sulfur-containing amino acid present in high concentrations in mammals, marine invertebrates, fish and marine algae. Taurine plays a role in physiological functions in these organisms such as antioxidation, cell cytotoxicity reduction, osmoregulation and membrane stabilisation [57, 58]. Some microorganisms utilise taurine as a sulfur source under sulfate starvation [56] and as a source for growth [59]. The high number of *TauD* genes identified suggests the utilisation of taurine as a nutrient source by *O. mirabilis*.

**Fig. 8:**
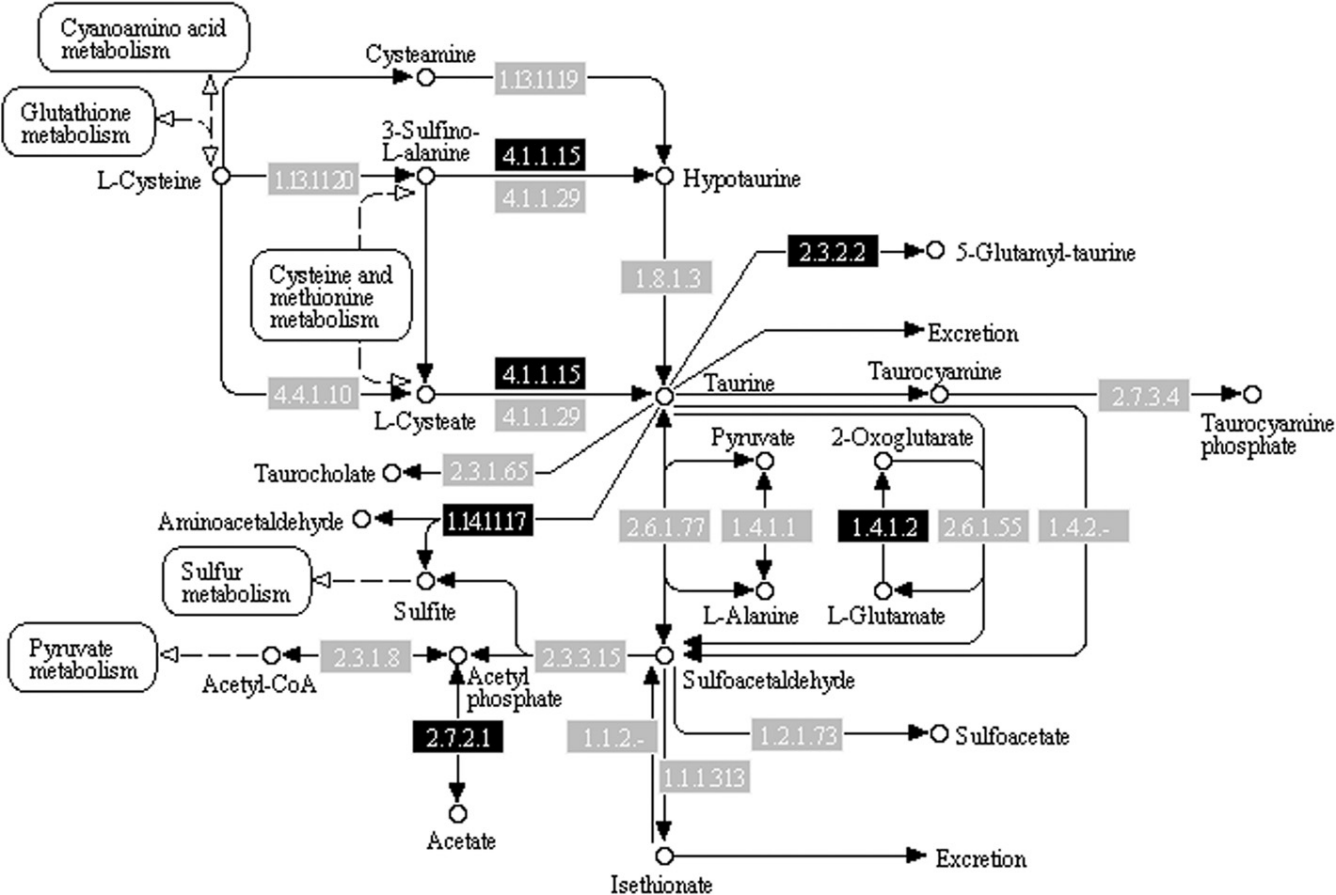
KEGG map of taurine and hypotaurine metabolism. Genes annotated via KEGG are shaded. Cysteine dioxygenase (EC 4.4.1.10) was not annotated. However, the intermediate product, L-cysteate may be supplied via the cysteine and methionine metabolism pathway

Recently, a Dothideomycetes, *Acidomyces richmondensis* was found able to synthesise and degrade taurine in a biofilm study. Taurine was then suggested to act as a compatible solute protecting the microbes from osmotic stress [60]. KEGG annotations showed that UM 578 might produce taurine (Fig. 8). Two genes (UM578_322 and UM578_7794) were mapped to the glutamate decarboxylase (EC 4.1.1.15) in the taurine metabolism pathway. In addition, we managed to identify a gene (UM578_7116) annotated as cysteine lyase that is 53.81 % identical to *Saccharomyces pombe* cysteine lyase [GenBank: O94350]. This completes the taurine metabolism pathway. Thus, *O. mirabilis* that is frequently isolated from immensely low water availability environments such as coastal hypersaline and bathroom surfaces [61], might also acquire taurine in osmoregulation.

Among other enriched Pfam domains, the glutathione-S-transferase domains encode enzymes well-known to be responsible for detoxification by catalysing the conjugation of glutathione to xenobiotics, pesticides and drugs [62]. The Pfam families encoding the N-terminal and C-terminal of glutathione-S-transferase increased from 15 to 30 and 11 to 20 copies respectively (Fig. 7c). RTA1-like protein that is also involved in detoxification has expanded from 12 to 21 copies (Fig. 7d). The RTA1-like protein plays a role in resistance to 7-aminocholesterol and prevents toxicity by binding to toxic substances [63]. The inflation of these Pfam families might contribute to the survival of *O. mirabilis* in domestic environments that are rich in xenobiotics and compounds toxic to the fungus.

## Conclusions

Our *in silico* genome analysis of *O. mirabilis* UM 578 revealed potential genes that enable the fungus to thrive in hostile environments and the involvement of mycotoxin production. Our analysis indicated that plant materials may not be the primary source of nutrient for this fungus. Occasional disease in humans may be related to the presence of several putative peptidases involved in the extracellular matrix and collagen degradation together with the action of lipases. The isolate might be heterothallic, and the mating activity remains to be elucidated. The expansion of genes involved in the degradation of taurine and detoxification enables the fungus to survive in the man-made hostile environment. This in-depth analysis of UM 578 genome provides a platform for more targeted functional studies in the future.

## Methods

### Fungal isolate sampling

*O. mirabilis* UM 578 was isolated from the skin scraping of a patient in the University of Malaya Medical Centre (UMMC), Malaysia. Morphological identification of the isolate was conducted as previously described [8]. The isolate was sub-cultured on Sabouraud Dextrose Agar (SDA, 10 g/L Mycological peptone, 40 g/L glucose and 15 g/L agar; Oxoid, UK). The 14 day-old culture was incubated at 30 °C and the fungal colony was observed. Slide culture was carried out to study the microscopic characteristics.

### Molecular identification

Molecular identification was conducted accordingly with DNA extraction, amplification of the internal transcribed spacer (ITS) region followed by DNA sequencing [8]. The identity of the fungal isolate was determined via BLASTn search against NCBI-nucleotide database. A total of 30 ITS sequences of all *Ochroconis* species together with a representative strain from a previous study (UM 314) [8] and two outgroup strains, *Scolecobasidium excentricum* and *Sympoventuria capensis* were obtained from GenBank to construct a phylogenetic tree. Multiple sequence alignment of the ITS sequences was performed using M-Coffee [64] and all aligned sequences were concatenated into a unique final alignment using T-Coffee. Bayesian tree analysis was conducted using MrBayes [65]. The analysis was carried out using reversible jump Markov Chain Monte Carlo (MCMC) averaging over general time reversible (GTR) rate model space and gamma-distributed rate heterogeneity for all subsets of partitioned scheme. The stationary state frequencies were fixed to be equal. A total of 500,000 generations were run with a sampling frequency of 100, and diagnostics were calculated for every 1000 generations. The first 1250 trees were discarded based on the burn-in setting of 25 %, and convergence was assessed according to the Draft MrBayes version 3.2.1 Manual [66]. Standard deviation of split frequencies below 0.01, potential scale reduction factor (PSRF) reasonably close to 1.0 for all parameters and, no obvious trend for the plot of the generation versus the log probability of the data (the log likelihood values) were observed.

### Genomic DNA extraction, sequencing and *de novo* assembly

A large-scale DNA extraction was conducted based on a modified method as described in Kuan et al. [67]. The sequencing and assembly of UM 578 genome were carried out as described previously [9]. The library was prepared using TruSeq v3 Reagent Kits (Illumina). The 5-kb Illumina sequenced read was then combined with the 500-bp Illumina sequenced read for further processing. Both sets of sequenced reads were pre-processed by trimming two and four bases from the 5’ end of 500-bp and 5-kb reads respectively. Bases with a Phred quality below Qv20 were trimmed from the 3’ end of the reads. The trimmed reads shorter than 50 bp and reads with 40 % bases having Qv ≤ 20 were filtered out using FASTX-Toolkit (http://hannonlab.cshl.edu/fastx_toolkit/). Substitution error correction of pre-processed sequencing reads was performed using Quake version 0.3.5 [68] with 16-mer setting. The error corrected reads were assembled using Velvet version 1.2.07 [69] with k-mer setting = 67, scaffolding = no, insert_length = 500, ins_length_sd = 10, insert_length2 = 5000, ins_length2_sd = 1400 and shortMatePaired2 = yes. Contig sequences assembled from the Velvet were further scaffolded using SSPACE Basic version 2.0 (parameters: −z = 100, −k = 5, −a = 0.3 and -n = 30) [70] and the GapFiller version 1.10 (parameters: −m = 60, −o = 15, −r = 0.8, −n = 30 and -T = 40) was used to perform gap filling by utilising paired-end information from both libraries [71].

### Gene prediction and annotation

Interspersed repetitive elements and low complexity DNA sequences were masked using RepeatMasker version open-3.3.0 with the Repbase fungal library version rm-20120418, followed by masking off the RNA sequences. The rRNAs were identified using RNAmmer version 1.2 [72] while tRNAs were detected by tRNAscan-SE version 1.3.1 [73]. Prediction of genes was carried out using GeneMark-ES version 2.3e [10]. The function of putative coding sequences (CDSs) was annotated via local BLAST searches against NCBI nr and SwissProt databases. Local BLAST2GO was also conducted to annotate GO and KEGG metabolic pathways [74]. KOG annotation were performed [75] and Interpro protein domain families match to Pfam database was performed using InterProScan 5 [76]. The GO annotations were plotted using WEGO [77]. Putative transposable elements were identified via PSI-TBLASTN search of the genome with a collection of (retro-) transposon ORF homology profiles from Transposon-PSI (http://transposonpsi.sourceforge.net).

Predicted protein models were submitted to dbCAN [78] for annotation of Carbohydrate-Active enZymes (CAZymes). A batch blast of UM 578 protein models against MEROPS database [79] was conducted for peptidases identification. The prediction of secreted proteins was carried out using the method of Ohm et al. [12]. SignalP version 4.1 [80] was used to predict the cleavage sites and signal peptide/non-signal peptide. The transmembrane (TM) domains were identified using TMHMM version 2.0 [81]. Secreted proteins were selected based on the presence of 40 amino acids at N-terminal as TMM and proteins without TM domains. Lipases were predicted by BLASTP search against the Lipase Engineering Database (LED) as previously described [12] together with six other fungi (Additional file 1: Table S7). Secondary metabolite backbone genes and associated genes for secondary metabolite biosynthesis cluster were predicted using web-based SMURF analysis tool [82]. The organisation of putative gene clusters were retrieved from the genome using sequence viewer Artemis version 12.0 [83].

### Orthologous genes and genome comparative analysis

The predicted proteomes of 17 publicly available fungal genomes were retrieved from several databases (Additional file 1: Table S1). Orthologues in UM 578 were determined by employing OrthoMCL version 2.0.9 [84]. Protein sequences ≥33 amino acids from all the genomes were clustered via all-against-all BLASTP searches. Orthologues were identified as protein sequences with reciprocal best blast hits from distinct genomes. OrthoMCL applies Markov Cluster algorithm [85] with 1e-5 BLASTP e-value cut-off and 1.5 inflation parameter.

### Phylogenomic analysis

A phylogenomic tree was constructed using predicted proteome clusters generated from the comparative analysis (Additional file 1: Table S1). ClustalW version 2.0 [86] was used to compile individual multiple sequence alignments for 917 single-copy orthologous genes. Spurious sequences or poorly aligned regions were removed using trimAL (with the *automated* option). A super-alignment with 357,792 characters was concatenated from all individually filtered alignments. Bayesian MCMC analysis was run with a burn-in setting of 25 % and sampling frequency of 100 for 100,000 generations. A mixed amino acid model with gamma-distributed rate variation across sites and a proportion of invariable sites were selected for the phylogenetic analysis.

### Gene families expansion and contraction analysis

The protein families of the 17 selected fungi were identified by Pfam analysis using pfam scan.pl search against the Pfam database. The database and tools were downloaded from Sanger Centre FTP site (ftp://ftp.sanger.ac.uk/pub/databases/Pfam/current release/ for database and ftp://ftp.sanger.ac.uk/pub/databases/Pfam/Tools/ for tools). Analysis of Pfam domain expansion and contraction was performed with CAFE software using a stochastic birth and death model [87]. The ultrametric phylogenomic tree and pfam protein domain families were used as input.

## Ethics approval

Fungal cultures are part of the routine management of infected patients in the Medical Centre and isolates are made anonymous before they are used for studies. As we were not involved in specimen collection and had no data traceable to the identity of the infected patient from whom UM578 was derived, ethical clearance for this study was exempted from the UMMC Medical Ethics Committee (http://umresearch.um.edu.my/doc/File/UMREC/6_CODE%20OF%20RESEARCH%20ETHICS%20%20IN%20UNIVERSITY%20OF%20MALAYA.pdf).

## Availability of data and material

The data sets supporting the results of this article are included within the article and its additional files. The nucleotide sequence of *O. mirabilis* UM 578 ITS region reported in this paper is available at DDBJ/EMBL/GenBank with accession number KP639587. The nucleotide sequence of *O. mirabilis* UM 578 genome reported in this manuscript is also available at DDBJ/EMBL/GenBank with accession number AZYM00000000. The version described in this paper is version AZYM01000000. The gene models reported can be accessed via in-house database, DemaDb (fungaldb.um.edu.my). The phylogenetic data of ITS-based phylogenetic and phylogenomic trees have been deposited in TreeBase (study number: S18646).

## Additional files

Additional file 1: Table S1. List of genome sequences used in gene families analysis and phylogenomic tree construction. **Table S2.** Number of CAZyme modules predicted in UM 578 genome. **Table S3.** Plant cell wall and fungal cell wall degrading and modifying CAZyme families predicted in UM 578. **Table S4.** Number of peptidases predicted in UM 578. **Table S5.** Comparison of putative secreted proteases families which had been reported to be expanded in dermatophytes. **Table S6.** Gene families clusters shared among UM 578 with *Trichophyton rubrum* and *T. verrucosum* in this study. **Table S7.** Predicted lipases in the genomes of UM 578 with skin-inhabiting and non-skin inhabiting fungi. **Table S8.** Putative biosynthetic pathway genes involved in aflatoxin (AF), sterigmatocystin (ST) and dothistromin (DOT) production predicted in UM 578. **Table S9.** Putative biosynthetic pathway genes for trichothecene production predicted in UM 578. **Table S10.** Predicted gene involved in sexual reproduction in UM 578. **Table S11.** UM 578 specific gene families clusters compared to 16 publicly available fungal genomes. **Table S12.** Expansion and contraction of Pfam families from CAFE analysis. The *P*-value for whole family expansion/ contraction is shown as family wide *P*-value and the node specific for UM 578 is shown (Additional file 2: Figure S15). Only families with family wide *P*-value ≤0.01 are shown. (XLS 307 kb) Additional file 2: Figure S1. KEGG map of styrene degradation. Genes annotated via KEGG are shaded. Z-phenylacetaldoxime degradation by nitrilase (EC 3.5.5.1), nitrile hydratase (EC 4.2.1.84) and amidase (EC 3.5.1.4). Although the phenylacetaldoxime dehydratase (EC 4.99.1.7) was not mapped, the gene was found in the genome. **Figure S2.** Alignment of putative phenylacetaldoxime dehydratase of *O. mirabilis* UM 578 (UM578_4049) with *Bacillus* sp. OxB-1 (P82604). Identical and similar residues are black and gray shaded respectively. The haem-containing dehydratase region is indicated by asterisk. **Figure S3.** Putative aldoxime-nitrile pathway gene cluster of UM 578. The phyenylacetaldoxime dehydratase (UM578_4049) and nitrilase (UM578_5050). The direction of transcription is indicated by the arrow for each gene. **Figure S4.** KEGG map of atrazine degradation. Genes annotated via KEGG are shaded. Cyanamide was degraded by cyanamide hydratase (EC 4.2.1.69) and urease (EC 35.1.5). **Figure S5.** Alignment of predicted metallopeptidase M14A of *O. mirabilis* UM 578 (UM578_1644). Alignment was carried out with metallopeptidase MeCPA from *Metarhizium anisopliae* (AAB68600) and TruMcpA from *Trichophyton rubrum* (ABW79919). Identical and similar residues are black and gray shaded, respectively. The zinc-binding residues are indicated by an asterisk. The active-site residues are indicated by circles. Conserved residues involved in substrate binding are indicated by solid triangles. The conserved Cys residues forming disulfide bridges are indicated by solid rhombus. **Figure S6.** Alignment of predicted serine carboxypeptidase of *O.mirabilis* UM 578 (UM578_13449). Alignment was carried out with TruSCPA from *Trichophyton rubrum* (AAS76667) and AfuCp1 from *Aspergillus fumigatus* (AAR91697). Identical and similar residues are black and gray shaded respectively. The consensus active residues are indicated by asterisk (Ser228, Asp439 and His497). **Figure S7.** Alignment of predicted leucine aminopeptidase (LAP) of *O. mirabilis* UM 578 (UM578_7056). Alignment was carried out with TruLAP1 from *Trichophyton rubrum* (AAS76670) and AfuLAP1 from *Aspergillus fumigatus* (AAR996058). Identical and similar residues are black and gray shaded respectively. The consensus binding sites for the first and the second Zn^2+^ ion binding sites are indicated in triangle (His180 and Asp265) and in rhombus (Glu238 and His347) respectively. The Asp199 is the residue bridging the two Zn^2+^ ions is indicated in circle. The active sites (Asp182 and Glu237) are indicated by asterisk. **Figure S8.** Alignment of predicted leucine aminopeptidase (LAP) of *O.mirabilis* UM 578 (UM578_5513). Alignment was carried out with TruLAP2 from *Trichophyton rubrum* (AAS76669) and AfuLAP2 from *Aspergillus fumigatus* (AAR96059). Identical and similar residues are black and gray shaded respectively. The consensus binding sites for the first and the second Zn^2+^ ion binding sites are indicated in triangle (His252 and Asp326) and in rhombus (Glu297 and His424) respectively. The Asp264 is the residue bridging the two Zn^2+^ ions is indicated in circle. The active sites (Asp254 and Glu296) are indicated in asterisk. **Figure S9.** Alignment of predicted dipeptidyl peptidase IV (DPPIV) of *O. mirabilis* UM 578 (UM578_9285). Alignment was carried out with TruDPPIV from *Trichophyton rubrum* (AAS76665) and AfuDPPIV from *Aspergillus fumigatus* (AAC34310). Identical and similar residues are black and gray shaded respectively. The catalytic triad is indicated in asterisk (Ser619, Asp 696, His731). **Figure S10.** Alignment of predicted dipeptidyl peptidase V (DPPV) of *O. mirabilis* UM 578 (UM578_9264). Alignment was carried out with TruDPPIV from *Trichophyton rubrum* (AAN03632) and AfuDPPIV from *Aspergillus fumigatus* (AAB67282). Identical and similar residues are black and gray shaded respectively. The catalytic triad is indicated in asterisk (Ser566, Asp647, His679). **Figure S11.** TMpred output of putative sulphite efflux pump (*ssu1*) in UM 578. The putative gene, UM578_9214 has ten membrane-spanning helixes and hydrophilic N- and C- termini. **Figure S12.** Putative melanin biosynthesis cluster in UM 578. The organisation and orientation of genes involved in melanin biosynthesis are similar to that of the reported melanin gene cluster in *C. heterostrophus* [GenBank: AAR90272] and *A. brassicicola* [GenBank: BAD22832]. The predicted genes encode polyketide synthase (UM578_2557), transcription factor Cmr1 (UM578_2558) and tetrahydroxynaphthalene reductase (UM578_2559). The direction of transcription is indicated by the arrow for each gene. **Figure S13.** Putative trichothecene biosynthesis cluster in UM 578. The 6751 bp cluster encompasses the trichodiene synthase (UM578_3030) with two cytochrome P450 encoding genes (UM578_3031 and UM578_3032) and the trichothecene efflux pump (UM578_3033). The direction of transcription is indicated by the arrow for each gene. **Figure S14.** Putative gene organisation of mating type genes in UM 578. The neighbouring genes of alpha-domain containing gene (UM578_3656) encompass the homeodomain-containing protein (UM578_3655), DNA lyase APN2 (UM578_3657) and cytochrome C oxidase Vla Cox13 (UM578_3658). The direction of transcription is indicated by the arrow for each gene. **Figure S15.** Phylogenomic tree showing number of each node in expansion/ contraction analysis. The number of genes and *P*-value for UM 578 (node 24) and the internode (node 23) are shown in Additional file 1: Table S12. (PDF 1000 kb)

## Abbreviations

AA: Auxiliary activities
ACP: Acyl carrier protein
AF: Aflatoxin
AT: Acyltransferase
CAZymes: Carbohydrate active enZymes
CBM: Carbohydrate-binding modules
CE: Carbohydrate esterases
DHN: 1,8-dihydroxynaphthalene
DMAT: Dimethylallyl tryptophan synthases
DPP: Dipeptidyl peptidase
GH: Glycoside hydrolases
GO: Gene ontology
GT: Glycosyltransferases
ITS: Internal transcribed spacer
KEGG: Kyoto encyclopedia of genes and genomes
KOG: EuKaryotic Orthologous Group
KS: Ketosynthase
LAP: Leucine aminopeptidase
MCMC: Markov chain Monte Carlo
NRPS: Nonribosomal peptide synthases
PKS: Polyketide synthases
PL: Polysaccharide lyases
SDA: Sabouraud Dextrose Agar
ST: Sterigmatocystin
TE: Thioesterase
