# Supplementary material for: The genome of newly classified Ochroconis mirabilis: Insights into fungal adaptation to different living conditions
Source: BMC Genomics. 2016 Feb 3;17:91. doi: 10.1186/s12864-016-2409-8 (PMC4738786; doi:10.1186/s12864-016-2409-8)
Supplement: Additional file 2: Figure S1. — KEGG map of styrene degradation. Genes annotated via KEGG are shaded. Z-phenylacetaldoxime degradation by nitrilase (EC 3.5.5.1), nitrile hydratase (EC 4.2.1.84) and amidase (EC 3.5.1.4). Although the phenylacetaldoxime dehydratase (EC 4.99.1.7) was not mapped, the gene was found in the genome. Figure S2. Alignment of putative phenylacetaldoxime dehydratase of O. mirabilis UM 578 (UM578_4049) with Bacillus sp. OxB-1 (P82604). Identical and similar residues are black and gray shaded respectively. The haem-containing dehydratase region is indicated by asterisk. Figure S3. Putative aldoxime-nitrile pathway gene cluster of UM 578. The phyenylacetaldoxime dehydratase (UM578_4049) and nitrilase (UM578_5050). The direction of transcription is indicated by the arrow for each gene. Figure S4. KEGG map of atrazine degradation. Genes annotated via KEGG are shaded. Cyanamide was degraded by cyanamide hydratase (EC 4.2.1.69) and urease (EC 35.1.5). Figure S5. Alignment of predicted metallopeptidase M14A of O. mirabilis UM 578 (UM578_1644). Alignment was carried out with metallopeptidase MeCPA from Metarhizium anisopliae (AAB68600) and TruMcpA from Trichophyton rubrum (ABW79919). Identical and similar residues are black and gray shaded, respectively. The zinc-binding residues are indicated by an asterisk. The active-site residues are indicated by circles. Conserved residues involved in substrate binding are indicated by solid triangles. The conserved Cys residues forming disulfide bridges are indicated by solid rhombus. Figure S6. Alignment of predicted serine carboxypeptidase of O.mirabilis UM 578 (UM578_13449). Alignment was carried out with TruSCPA from Trichophyton rubrum (AAS76667) and AfuCp1 from Aspergillus fumigatus (AAR91697). Identical and similar residues are black and gray shaded respectively. The consensus active residues are indicated by asterisk (Ser228, Asp439 and His497). Figure S7. Alignment of predicted leucine aminopeptidase (LAP) of O. mirabilis UM 578 (UM578 [file 12864_2016_2409_MOESM2_ESM.pdf]

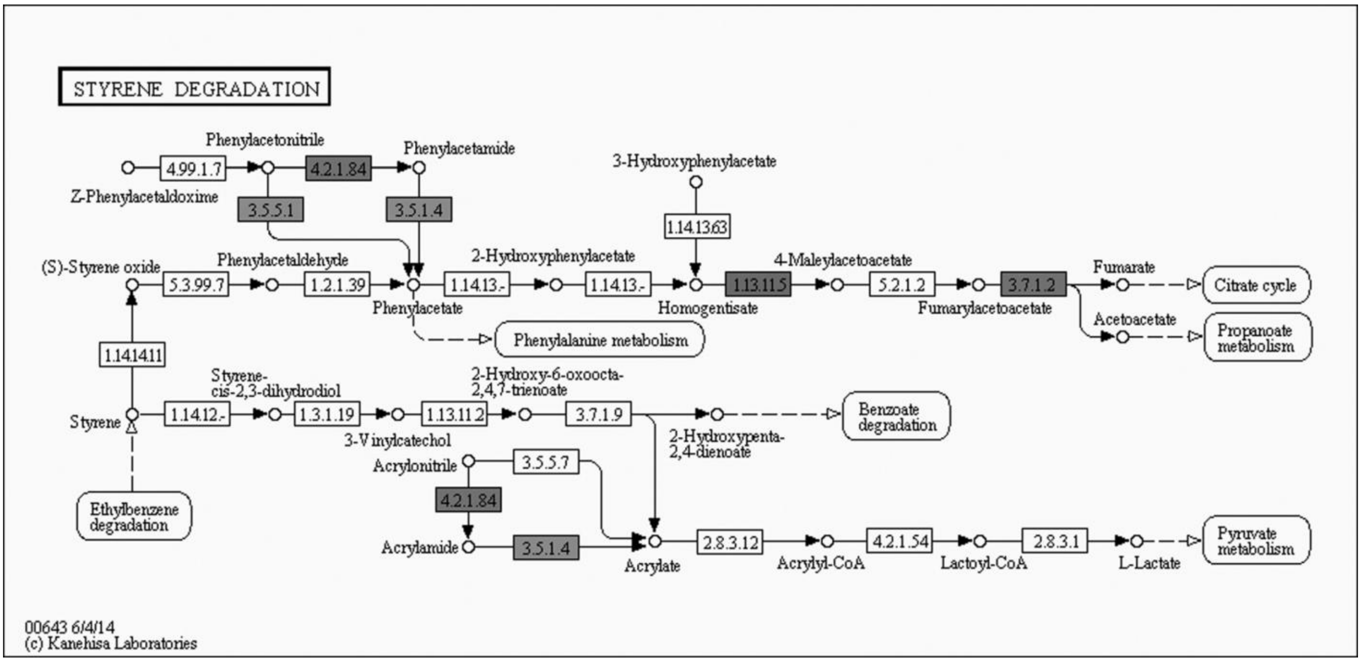

Fig. S1 KEGG map of styrene degradation. Genes annotated via KEGG are shaded. Z-phenylacetaldoxime degradation by nitrilase (EC 3.5.5.1), nitrile hydratase (EC 4.2.1.84) and amidase (EC 3.5.1.4). Although the phenylacetaldoxime dehydratase (EC 4.99.1.7) was not mapped, the gene was found in the genome.

```
                                                                 ***************
UM578_4049     1 MELESAIPQHLQTERSLPSSLPSKWQPPFPAYIARWPKSAKGLVMAIIGAQHQSRPCNDS
P82604_BOXD    1 -----------------MKNMPENHNPQANAWTAEFPPEMSYVVFAQIGIQSKS---LDH

                 ************************************************************
UM578_4049    61 SSQRLISFMEARSADNKPKYWELASYTDNQNHYNEAIFAYWPTREAYEAWSSSSEFEKWW
P82604_BOXD   41 AAEHLGMMKKSFDLRTGPKHVDRALHQGADGYQDSIFLAYWDEPETFKSWVADPEVQKWW

                 ************************************************************
UM578_4049   121 HGLDAATEQ-NGWFKEVFFPAIDHFETVFSNNAANEGAAHMKKEMSGPIAEHVYWGSMRD
P82604_BOXD  101 SGKKIDENSPIGYWSEVTTIPIDHFETLHSGENYDNGVSHFVPIKH--TEVHEYWGAMRD

                 ************************************************************
UM578_4049   180 RLPASQHDQMTGGVWQCASGADGSAAQSRETKRKRVRVPGHQNLAVIRSGQDWSDTLPNE
P82604_BOXD  159 RMPVSASSDLES-----PLGLQLPEPIVRESFGKRLKVTAPDNICLIRTAQNWSKCGSGE

                 ************************************************************
UM578_4049   240 RKLYLETMHPVLTKGMDFLRDHGSEVGCISNRFMTVTESKAPARNTDKTFGLGYFDDLAS
P82604_BOXD  214 RETYIGLVEPTLIKANTFLRENASETGCISS--KLVYEQTHDGEIVDKSCVIGYYLSMGH

                 **********************************************************
UM578_4049   300 LEKWSKQHKTHLDIFGGFLKYAKELQNDISLKLFHEVLVLGSDQQDLEYMNCHPGTGMLP
P82604_BOXD  272 LERWTHDHPTHKAIYGTFYEMLKRHDFKTELALWHEVSVLQSKDIELIYVNCHPSTGFLP

UM578_4049   360 ALQR----------------
P82604_BOXD  332 FFEVTEIQEPLLKSPSVRIQ
```

Fig. S2 Alignment of putative phenylacetaldoxime dehydratase of *O. mirabilis* UM 578 (UM578_4049) with *Bacillus* sp. OxB-1 (P82604). Identical and similar residues are black and gray shaded respectively. The haem-containing dehydratase region is indicated by asterisk.

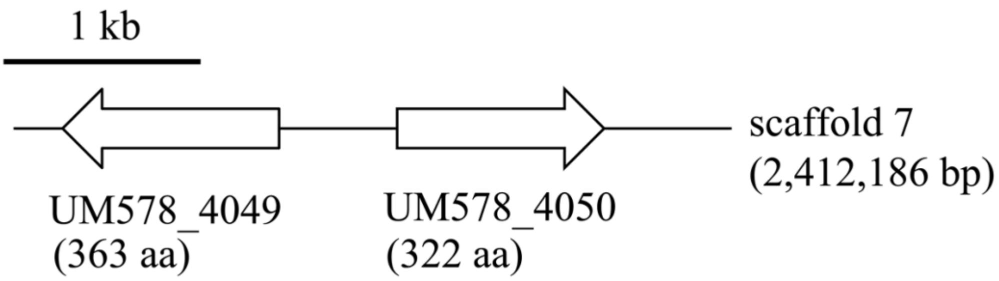

Fig. S3 Putative aldoxime-nitrile pathway gene cluster of UM 578. The phyenylacetaldoxime dehydratase (UM578_4049) and nitrilase (UM578_5050). The direction of transcription is indicated by the arrow for each gene.

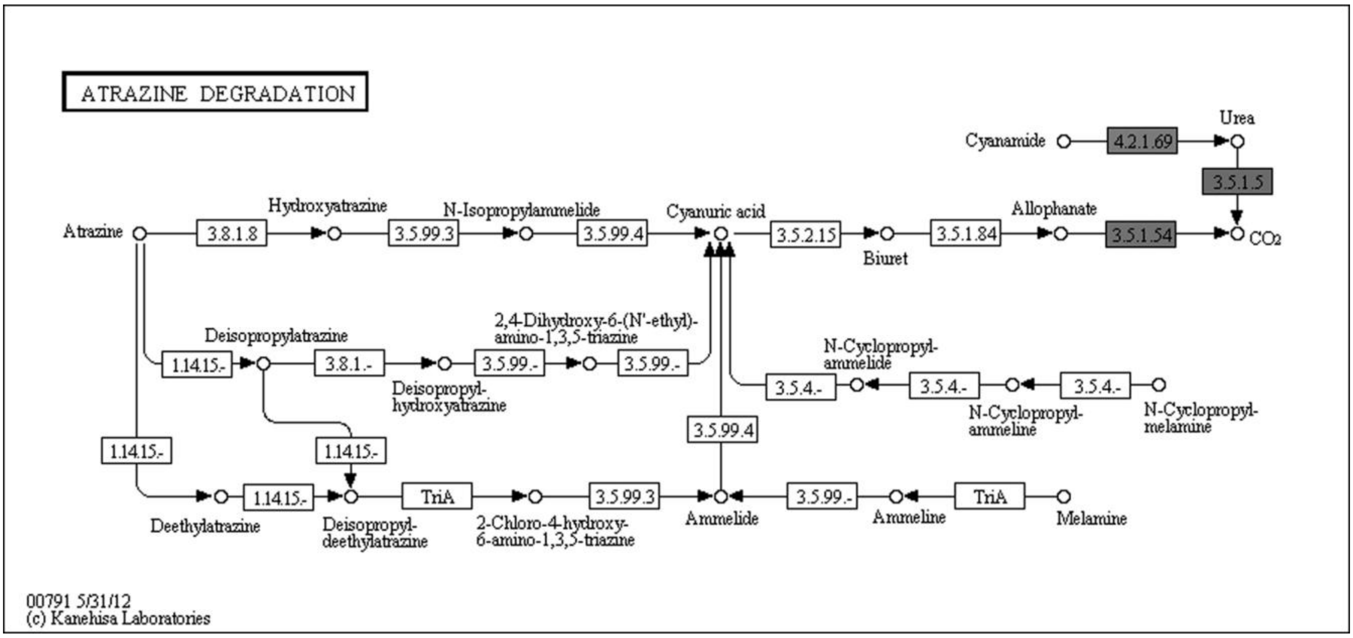

Fig. S4 KEGG map of atrazine degradation. Genes annotated via KEGG are shaded. Cyanamide was degraded by cyanamide hydratase (EC 4.2.1.69) and urease (EC 35.1.5).

```
UM578_1644          1 MKWLASVLALCIGTAVAAPADGTVSYAGYQVVRIN---GDVAASKAKLGSLTYDQWH---
MeCPA_AAB68600      1 MRVVAF-FACVVATAATMPAESPVSYDGYKVFRVPVVDDG-THIQSLIDHLNLNVWQPPS
TruMcpA_ABW79919    1 MRSVLS-LALLAANVVTAAVVSPFDYSGYKVIRVPTQKDNVKEVQRVITDLNLDTWKYPK

UM578_1644         55 QEGSILDIALPADQVDAFKSL--GLDYSVMHEDLGSSIEAESGLRASFDNVKLANAAVNT
MeCPA_AAB68600     59 KKGAFADIQVAPSQLAAFENAMKGRSFEIMHEDLGDSIAREGTI----QAY-AAGS-ANA
TruMcpA_ABW79919   60 SEGQNADIVVPPSQISSFMERISGMNIEMMHEDLGLSIRNETSF----EAY-SAGYAPDI

UM578_1644        113 TWFSAYHPWAEHQSYLRDLQARFPNQSALVSTGRSYQGRDMFGIHLWGASGPG-KPAVLW
MeCPA_AAB68600    113 SWFTSYHPYNDHLQWMKDIAFQYPSNVKSVTSGTTGDGNTITGLHIFGSSGGGNKPAVVF
TruMcpA_ABW79919  115 NWFKSYHSYQDHLSYLQDLQGLFRTRSEYVDAGKSHEGRTIPALHIWGSGGKNSKPAIIF

                          *  *
UM578_1644        172 HGTVHAREWISAMVVEYLTEQLVTTYGSDATTKSILDSYDFHIFPFVNPDGFVYSQTTNR
MeCPA_AAB68600    173 HGTVHAREWIVAMTLEYITNELLAKYATDSAVKAVVDKYDFYMFPIVNVDGFKYTQSSDR
TruMcpA_ABW79919  175 HGTIHAREWITTMVTEYMAWSFLSQYNKNADITSIVDNFDIWIFPIVNPDGFAFTQTSNR

                        •           ♦      ▼                 ♦
UM578_1644        232 MWRKNRQPGPSGSRCVGRDINRNWPAEWDSNPDGASPDPCAETYRGQAPSDSPENQGLVS
MeCPA_AAB68600    233 MWRKNRSRNQ-GSSCLGTDPNRNWPYKWDG-P-GSSTNPCTETYRGASAGNSPEVKSYIA
TruMcpA_ABW79919  235 LWRKNRQPNP-NARCPGRDLNRNYPYQWVG-P-GSSSNPCSDTYRGAQPGDGTEIKVHIA

                                       * ▼           ♦
UM578_1644        292 YVNKLRDAAGIKLYIDWHSYGQYILSPFGYTCDVLPQDNNEHQVLMQKTAAAIRASKGKT
MeCPA_AAB68600    290 FLDKIKKSQGVKLYIDWHSYSQLFMTPYGYSCSARTPNNAALQALAKGASDAMRSVHGTT
TruMcpA_ABW79919  292 NMKKIAANKGIAMFVDWHSYGQLFMSPYGYSCTARPPTDARHQELSRIFAQALKAVHGTP

                            ♦   ▼                      •
UM578_1644        352 FTYGPSCATLYATTGDSTDYLHGPGRSDYSITIELRDQGASGFVLPATQILPSGEEQWAG
MeCPA_AAB68600    350 FAYGPVCNVIYQVAGGSIDWVQDVLKADNVFTIELRDKGRYGFVLPPDQIIPSGEESFAG
TruMcpA_ABW79919  352 YKTGPICNTIYQVNGDSVDYALEVLKVKLSLTAELRDTGARGFVLPADQIIPSGEETLAG

UM578_1644        412 MKVMLTS---M
MeCPA_AAB68600    410 AMHLFQQM--S
TruMcpA_ABW79919  412 TVAMLKAVIQG
```

Fig. S5 Alignment of predicted metallopeptidase M14A of *O. mirabilis* UM 578 (UM578_1644). Alignment was carried out with metallopeptidase MeCPA from *Metarhizium anisopliae* (AAB68600) and TruMcpA from *Trichophyton rubrum* (ABW79919). Identical and similar residues are black and gray shaded, respectively. The zinc-binding residues are indicated by an asterisk. The active-site residues are indicated by circles. Conserved residues involved in substrate binding are indicated by solid triangles. The conserved Cys residues forming disulfide bridges are indicated by solid rhombus.

```
UM578_13449            1 M-DLFIHMLFFLTFFMRASSASFPPTPQDITTIPSKKFSGRSISYKQTYICETTPGVKSF
AAS76667_TruSCPA       1 M-RFAASIAVALPVIHAASAQGFPPPVKGVTVVKSKFDENVKITYKENDICETTQGVRSF
AAR91697_AfuCp1        1 MLSLVTLLSGTAGLALTASAQYFPPTPEGLKVVHSKHQEGVKISYKEPGICETTPGVKSY

UM578_13449           60 SGYVNLPAD-E---SLGRASSTSIYFWYFEARNDPKNAPLAMWLQGGPGVPSITAAVGEN
AAS76667_TruSCPA      60 TGHVHLPPD-NDDFGVYRNYSINTFFWFFEAREDPKNAPLSIWLNGGPGSSSMIGLFQEN
AAR91697_AfuCp1       61 SGYVHLPPGTLNDVDVDQQYPINTFFCFFESRNDPIHAPLAIWMNGGPGSSSMIGLLQEN

UM578_13449          116 GPCLVMDDSKSTKLNPWSWNEEANMLYIDQPVQAGFSYNNLVNGTLNELPLPFIFEPANF
AAS76667_TruSCPA     119 GPCWVNEDSKSTTNNSFSWNNKVNMLYIDQPNQVGFSYDVPTNITYSTINDTI--SVADF
AAR91697_AfuCp1      121 GPCLVNADSNSTEINPWSWNNYVNMLYIDQPNQVGFSYDVPTNGTYNQLTTAW--NVSAF

UM578_13449          176 SKVGVPETNLTFLTGTFAEPDIGGAPNTTIAAAPVI----------FPEHHATRNRFSIW
AAS76667_TruSCPA     177 SNG-VPAQNLSTLVGTGSSQNPWATANNTVNAARSIWHFAQVWFQEFPEHKPNNNKISIW
AAR91697_AfuCp1      179 PDGKVPEQNNTFYVGTFPSMNRTATANTTQNAARSLWHFAQTWFSEFPEYKPHDDRVSIW

                          *
UM578_13449          226 AESYGGHYAPVYANYFEEQNDRIAQGKLR--GPAKPIHVDTVGLLNACIDIDTQMPFYPE
AAS76667_TruSCPA     236 TESYGGRYGPSFASYFQEQNEKIKNHTITEEGEMHILNLDTLGIINGCIDLMFQAESYAE
AAR91697_AfuCp1      239 TESYGGRYGPSFAAFFQEQNEKIEEGALP--DEYHYIHLDTLGIINGCVDLLTQAPFYPD

UM578_13449          284 FAFNNTYGIKAINESQYKAALAA---ADDCRELTTECRKLASEQDPMGRGNNPDVNDACL
AAS76667_TruSCPA     296 FPYNNTYGIKAYTKEKRDAILHDIHRPDGCFDKVTKCREAAKEGDPHFYSNNATVNTICA
AAR91697_AfuCp1      297 MAYNNTYGIEAINKTVYERAMNAWSKPGGCKDLIVKCRELAAEGDPTMSGHNETVNEACR

UM578_13449          341 EAYLFCFSKMHSGYDK--SRNLFDIASPVLESFPPKWPAGYFNTPEIQQALGVPLNFTGN
AAS76667_TruSCPA     356 DANSACDKYLMDPFQE-TNLGYYDIAHPLQDPFPPPFYKGFLSQSSVLSDMGSPVNFSQY
AAR91697_AfuCp1      357 RANDYCSNQVEGPYILFSKRGYYDIAHFDPDPFPPPYFQGFLNQNWVQAALGVPVNFSIS

                                                            *
UM578_13449          399 SMVIASTFNLTGDFVRGDS--L-LNLGTLLDKGTNVAMVYGDRDYQCNWLGGEAISTSID
AAS76667_TruSCPA     415 AQAVGKSFHGVGDYARPDVRGFTGDIAYLLESGVKVALVYGDRDYICNWFGGEQVSLGLN
AAR91697_AfuCp1      417 VDSTYSAFASTGDYPRADVHGYLEDLAYVLDSGIKVALVYGDRDYACPWNGGEEVSLRVN

                                                          *
UM578_13449          456 WKASSKFSQAGYAELRTNASYVGGLVRQFGNLSFARVYQAGHEVPYYQPETAQQIFNRVM
AAS76667_TruSCPA     475 YTGTQDFHRAKYADVKVNSSYVGGVVRQHGNFSFTRVFEAGHEVPGYQPETALKIFERIM
AAR91697_AfuCp1      477 YSDSQSFQKAGYAPVQTNSSYIGGRVRQYGNFSFTRVFEAGHEVPAYQPQTAYEIFHRAL

UM578_13449          516 SKRDVAAGKDNLH--GGYHTTGRQSAFT-PSGMPPHEDPPRCYVWDVFETCTAEEQKVLA
AAS76667_TruSCPA     535 FNKDISTGEIDIAQKPDYGTTGTESTFHIKNDIPPSPEP-TCYLLSADGTCTPEQLNAIK
AAR91697_AfuCp1      537 FNRDIATGKMSLLKNATYASEGPSSTWEFKNEVPESPEP-TCYIQSLQSSCTEEQIQSVV

UM578_13449          573 NGTAVTENFIVVGYLAADGS------------------------TILFNSTNAV----Q
AAS76667_TruSCPA     594 DGTAVVENYIIKSPAASKGNPPPTTTSSPTAAPTAGSAMLKAPVAMLAISALTVLAFFL
AAR91697_AfuCp1      596 NGTALIKDWIVVEKVDI-----------------------------------------Y
```

Fig. S6 Alignment of predicted serine carboxypeptidase of *O.mirabilis* UM 578 (UM578_13449). Alignment was carried out with TruSCPA from *Trichophyton rubrum* (AAS76667) and AfuCp1 from *Aspergillus fumigatus* (AAR91697). Identical and similar residues are black and gray shaded respectively. The consensus active residues are indicated by asterisk (Ser228, Asp439 and His497).

```
UM578_7056            1 MKFASVLALGAAT--SVLASV-PN-HLQQAP--LINKEKFLIELGPGETRWITEDEKWEL
AAR96058_AfuLAP1      1 MKVLTAIALSAIAFTGAVAAVITQEAFLNNPRIHHDQEKYLIELAPYRTRWVTEEEKWAL
AAS76670_TruLAP1      1 MKLLSVLALSATAT-SVLGASIPV-DA--------RAEKFLIELAPGETRWVTEEEKWEL

UM578_7056           55 KRNGINFMDISDTQDLGM-S-MRSRATVKYPHSVAHNKSVSSLLPKLEKKNMRSHLETFT
AAR96058_AfuLAP1     61 KLDGVNFIDITEEHNTGFYPTLHSASYVKYPPKMQYAEEVAALNKNLSKENMKANLERFT
AAS76670_TruLAP1     51 KRKGQDFFDITDE-EVGFTAAVAQPAIA-YPTSIRHANAVNAMIATLSKENMQRDLTKLS

UM578_7056          113 SFHTRYYKSQYGAQSSAWLLSQVQQILGESGAMKHGARAVPFQHPWGQSSIIATIPGKSN
AAR96058_AfuLAP1    121 SFHTRYYKSQTGIRSATWLFDQVQRVVSESGAAEYGATVERFSHPWGQFSIIARIPGRTN
AAS76670_TruLAP1    109 SFQTAYYKVDFGKQSATWLQEQVQAAINTAGANRYGAKVASFRHNFAQHSIIATIPGRSP

                               ▼                  •
UM578_7056          173 KTVVIGAHQDSINLFLPSILAAPGADDDGSGTVTILEALRVLLTDEDTISGKAPNTIEFH
AAR96058_AfuLAP1    181 KTVVLGAHQDSINLFLPSILAAPGADDDGSGTVTILEALRGLLQSDAIAKGNASNTVEFH
AAS76670_TruLAP1    169 EVVVVGAHQDSINQRSPMTGRAPGADDNGSGSVTILEALRGVLRDQTILQGKAANTIEFH

                             ◆                          ▼
UM578_7056          233 WYSAEEGGLLGSQAIFQSYEKAGRDIKAMLQQDMTGYVQKTLDAGEPESVGVITDYVDPG
AAR96058_AfuLAP1    241 WYSAEEGGMLGSQAIFSNYKRNRREIKAMLQQDMTGYVQGALNAGVEEAIGIMVDYVDQG
AAS76670_TruLAP1    229 WYAGEEAGLLGSQAIFANYKQTGKKVKGMLNQDMTGYIKGMVDKGLKVSFGIITDNVNAN

                                                                  ◆
UM578_7056          293 LTNFIKEVITAYCEIPYIETKCGYACSDHASASKAGYPSAFVIESDFKYSDSKIHTTEDK
AAR96058_AfuLAP1    301 LTQFLKDVVTAYCSVGYLETKCGYACSDHTSASKYGYPAAMATEAEMENTNKKIHTTDDK
AAS76670_TruLAP1    289 LTKFVRMVITKYCSIPTIDTRCGYACSDHASANRNGYPSAMVAESPIDLLDPHLHTDSDN

UM578_7056          353 IEYLSFDHMLQHARLTLGLVYELAWAKFD
AAR96058_AfuLAP1    361 IKYLSFDHMLEHAKLSLGFAFELAFAPF-
AAS76670_TruLAP1    349 ISYLDFDHMIEHAKLIVGFVTELAK----
```

Fig. S7 Alignment of predicted leucine aminopeptidase (LAP) of *O. mirabilis* UM 578 (UM578_7056). Alignment was carried out with TruLAP1 from *Trichophyton rubrum* (AAS76670) and AfuLAP1 from *Aspergillus fumigatus* (AAR996058). Identical and similar residues are black and gray shaded respectively. The consensus binding sites for the first and the second Zn^{2+} ion binding sites are indicated in triangle (His180 and Asp265) and in rhombus (Glu238 and His347) respectively. The Asp199 is the residue bridging the two Zn^{2+} ions is indicated in circle. The active sites (Asp182 and Glu237) are indicated by asterisk.

```
UM578_5513           1 MKGAVLLG---A-STCLACVSAQKYGQPQTPPVTSEELQALLSTEDLLKGAQQLQDFANA
AAR96059_AfuLAP2     1 MKLLY------LTSFA-SLAVANGPGWDWKPRVHPKVLPQMIHLWDLLQGAQQLEDFAYA
AAS76669_TruLAP2     1 MKSQLLSLAVAVTTISQGVVGQEPFGWPFKPMVTQDDLQNKIKLKDIMAGVEKLQSFSDA

UM578_5513          57 N-GGNRVFGSPGHNATVNWLADTLRATGYYDVELQPFVALFSGGSAELTAAGSPVEAELL
AAR96059_AfuLAP2    54 YPERNRVFGGRAHEDTVNYLYRELKKTGYYDVYKQPQVHQWTRADQALTVDGQSYDATTM
AAS76669_TruLAP2    61 HPEKNRVFGGNGHKDTVEWIYNEIKATGYYDVKKQEQVHLWSHAEAALNANGKDLKASAM

UM578_5513         116 TYTPNGD-VAGPIVAAANLGCDAADFPPETTGAISLISRGTCPFAQKAANALTAGAIGAV
AAR96059_AfuLAP2   114 TYSPSVN-ATAPLAVVNNLGCVEADYPADLTGKIALISRGECTFATKSVLSAKAGAAAAL
AAS76669_TruLAP2   121 SYSPPASKIMAELVVAKNNGCNATDYPANTQGKIVLVERGVCSFGEKSAQAGDAKAAGAI

UM578_5513         175 VYNNVPGNLAGTLGGP----GDYPPTVGVTQEAGEAILAQIQAGAVEGTLLTNVIEENRT
AAR96059_AfuLAP2   173 VYNNIEGSMAGTLGGATSELGAYAPIAGISLADGQALIQMIQAGTVTANLWIDSQVENRT
AAS76669_TruLAP2   181 VYNNVPGSLAGTLGGL---DKRHVPTAGLSQEDGKNLATLVASGKIDVTMNVISLFENRT

                                            ▼ *         •
UM578_5513         231 TYNVLAETKGGDKQNVVMAGGHSDSVEAGPGINDDGSGIVGILNVALALTKFSVKNAVRF
AAR96059_AfuLAP2   233 TYNVIAQTKGGDPNNVVALGGHTDSVEAGPGINDDGSGIISNLVVAKALTRFSVKNAVRF
AAS76669_TruLAP2   238 TWNVIAETKGGDHNNVIMLGAHSDSVDAGPGINDNGSGSIGIMTVAKALTNFKLNNAVRF

                            *♦                            ▼
UM578_5513         291 GFWSAEEFGLLGSEHYMTVVNATAEIEKIRLYLNFDMIASPNYIYGIYDGDGNAFNISGP
AAR96059_AfuLAP2   293 CFWTAEEFGLLGSNYYVNSLNAT-EQAKIRLYLNFDMIASPNYALMIYDGDGSAFNLTGP
AAS76669_TruLAP2   298 AWWTAEEFGLLGSTFYVNSLDDR-ELHKVKLYLNFDMIGSPNFANQIYDGDGSAYNMTGP

UM578_5513         351 PGSAQIEATFENFFKTAGLNSVPTAFTGRSDYGPFLDNNIPAGGLFTGAEVVKTEEEVAL
AAR96059_AfuLAP2   352 AGSAQIERLFEDYYTSIRKPFVPTEFNGRSDYQAFILNGIPAGGLFTGAEAIKTEEQAQL
AAS76669_TruLAP2   357 AGSAEIEYLFEKFFDDQGIPHQPTAFTGRSDYSAFIKRNVPAGGLFTGAEVVKTPEQVKL

                                    ♦
UM578_5513         411 FGGTAGVAYDENYHEAGDTIDNLNLEAFLVNTKAIADSVARFAENLDDIPPVSRPNKFRR
AAR96059_AfuLAP2   412 FGGQAGVALDANYHAKGDNMTNLNREAFLINSRATAFAVATYANSLDSIPPRNMTTVVKR
AAS76669_TruLAP2   417 FGGEAGVAYDKNYHRKGDTVANINKGAIFLNTRAIAYAIAEYARSLKGFPTRPKTG--K-

UM578_5513         471 AYME-TLKKFRRRAVET-LRHGRDACGKHEHSEI
AAR96059_AfuLAP2   472 SQLE-QAM--K-RTPHTHTGGTG--CYKDRVE-Q
AAS76669_TruLAP2   474 RDVNPQYSKMP---------GGG--CGHHTVF-M
```

Fig. S8 Alignment of predicted leucine aminopeptidase (LAP) of *O.mirabilis* UM 578 (UM578_5513). Alignment was carried out with TruLAP2 from *Trichophyton rubrum* (AAS76669) and AfuLAP2 from *Aspergillus fumigatus* (AAR96059). Identical and similar residues are black and gray shaded respectively. The consensus binding sites for the first and the second Zn^{2+} ion binding sites are indicated in triangle (His252 and Asp326) and in rhombus (Glu297 and His424) respectively. The Asp264 is the residue bridging the two Zn^{2+} ions is indicated in circle. The active sites (Asp254 and Glu296) are indicated by asterisk.

```
UM578_9285           1 MRWLAYGLLACAAEAAVDKIIRPPTQPTGNGSKLLTFQEATTGK-LAARSSTFAWIAAEG
AAS76665_TruDPPIV    1 MKLLSLLMLAGIAQAIV--PPREPRSPTGGGNKLLTYKECVPRATISPRSISLAWINSEE
AAC34310_AfuDPPIV    1 MKW-SILLLVGCAAAID--VPRQPYAPTGSGKKRLTFNETVVKRAISPSAISVEWISTSE

UM578_9285          60 REGYYVTTANGSLVERNIVTQDQRTLVPAEQVPADYYEFWFNADATQILWAIDYTKQYRH
AAS76665_TruDPPIV   59 DGRYISQSDDGALILQNIVTNTNKTLVAADKVPKGYYDYWFKPDLSAVLWATNYTKQYRH
AAC34310_AfuDPPIV   58 DGDYVYQDQDGSLKIQSIVTNHTQTLVPADKVPEDAYSYWIHPNLSSVLWATNYTKQYRH

UM578_9285         120 SYFANYLVQDVASGEVSPLIPEQDGDIQYAVWSPSATDNTIAFVRGNNVYVWKNGTTTQI
AAS76665_TruDPPIV  119 SYFANYFILDIKKGSLTPLAQDQAGDIQYAQWSPM--NNSIAYVRXNDLYIWNNGKTKRI
AAC34310_AfuDPPIV  118 SYFADYFIQDVQSMKLRPLAPDQSGDIQYAQWSPT--GDAIAFVRGNNVFVWTNASTSQI

UM578_9285         180 TNDGSPDLFNGVPDWVYEEEIFGTNFVLWFNAAGDQLCYLTTNETGVPTFTVQYWMNHTE
AAS76665_TruDPPIV  177 TENGGPDIFNGVPDWVYEEEIFGDRFALWFSPDGEYLAYLRFNETGVPTYTIPYYKN---
AAC34310_AfuDPPIV  176 TNDGGPDLFNGVPDWIYEEEILGDRFALWFSPDGAYLAFLRFNETGVPTFTVPYYMD---

UM578_9285         240 PSESVALSYPDELDLRYPKVGATNPTIAANLLDLNWGAEGPAPKTLPVSGFDASDLIIGE
AAS76665_TruDPPIV  234 -KQKIAPAYPRELEIRYPKVSAKNPTVQFHLLNIASSQ----ETTIPVTAFPENDLVIGE
AAC34310_AfuDPPIV  233 -NEEIAPPYPRELELRYPKVSQTNPTVELNLLELRTGE----RTPVPIDAFDAKELIIGE

UM578_9285         300 VAWLDTQ---IAIRIFNRVQSMSKLYLYDTATEASQVTREQDGSDGWLDNHLAISYIGD-
AAS76665_TruDPPIV  289 VAWLSSGHDSVAYRAFNRVQDREKIVSVKVESKESKVIRERDGTDGWIDNLLSMSYIGNV
AAC34310_AfuDPPIV  288 VAWLTGKHDVVAVKAFNRVQDRQKVVAVDVASLRSKTISERDGTDGWLDNLLSMAYIGPI

UM578_9285         356 -G--Q--FVDQNDLSGYDHFYLYSVNDTASPVALTKGDFEVRSLLYVDQARRIAYYTSTE
AAS76665_TruDPPIV  349 NG--KEYYVDISDASGWAHIYLYPVD-GGKEIALTKGEWEVVAILKVDTKKKLIYFTSTK
AAC34310_AfuDPPIV  348 GESKEEYYIDISDQSGWAHLWLFPVA-GGEPIALTKGEWEVTNILSIDKPRQLVYFLSTK

UM578_9285         411 VHPTESHVFGVSLDTAESFNLTDTSLPGFWSASFTQEGNYYVLSYSGPNVPFQQLYAAND
AAS76665_TruDPPIV  406 YHSTTRHVYSVSYDTKVMTPLVNDKEAAYYTASFSAKGGYYILSYQGPNVPYQELYSTKD
AAC34310_AfuDPPIV  407 HHSTERHLYSVSWKTKEITPLVDDTVPAVWSASFSSQGGYYILSYRGPDVPYQDLYAIN-

UM578_9285         471 TATPIRTITDNAALIGNLSDYKLPVIEYLDLESPSGFTFSGMLRYPANFDASKKYPILFI
AAS76665_TruDPPIV  466 SKKPLKTITSNDALLEKLKEYKLPKVSFFEIKLPSGETLNVKQRLPPNFNPHKKYPVLFT
AAC34310_AfuDPPIV  466 STAPLRTITSNAAVLNALKEYTLPNITYFELALPSGETLNVMQRLPVKFSPKKKYPVLFT

UM578_9285         531 PYGGPGAQEVTKRMSAFNFKSYIAADTELEYITFTLDGRGTGFRGRAHRASVNRHLGEFE
AAS76665_TruDPPIV  526 PYGGPGAQEVSQAWNSLDFKSYITSDPELEYVTWTVDNRGTGYKGRKFRSAVAKRLGFLE
AAC34310_AfuDPPIV  526 PYGGPGAQEVSKPWQALDFKAYIASDPELEYITWTVDNRGTGYKGRAFRCQVASRLGELE

                                                   *
UM578_9285         591 AEDQIWAAQELSRRYNFIDTDKIQIWGWSFGGYLTAKVVEADSGVFSQGLSTAPVSDWRL
AAS76665_TruDPPIV  586 AQDQVFAAKEVLK-NRWADKDHIGIWGXSYGGFLTAKTLETDSGVFTFGISTAPVSDFRL
AAC34310_AfuDPPIV  586 AADQVFAAQQAAK-LPYVDAQHIAIWGWSYGGYLTGKVIETDSGAFSLGVQTAPVSDWRF

                                                                    *
UM578_9285         651 YDSMYTERYMGLPDSNFEGYNGTAVRKVDGFKDIAGGVLIQHGTGDDNVHFQNSEVLVDV
AAS76665_TruDPPIV  645 YDSMYTERYMKTVELNADGYSETAVHKVDGFKNLKGHYLIQHGTGDDNVHFQNAAVLSNT
AAC34310_AfuDPPIV  645 YDSMYTERYMKTLESNAAGYNASAIRKVAGYKNVRGGVLIQHGTGDDNVHFQNAAALVDT

                                           *
UM578_9285         711 LVGGGVGPDKLEVAYFTDSDHSIVYNGANTYVYQQLAERLFAEKIREVGPAPSHQWTATS
AAS76665_TruDPPIV  705 LMNGGVTADKLTTQWFTDSDHGIRYDMDSTYQYKQLSKMVYDQKQRRPESPPMHQWSKRV
AAC34310_AfuDPPIV  705 LVGAGVTPEKLQVQWFTDSDHGIRYHGGNVFLYRQLSKRLYEEKKRKE-KGEAHQWSKKS
```

```
UM578_9285         771 EQPLAKSKRDIKVAKRDQAKREAIDSRIGKFRRSLEV
AAS76665_TruDPPIV  765 L----------AALFGERAE----------------E
AAC34310_AfuDPPIV  764 V-----------------------------------L
```

Fig. S9 Alignment of predicted dipeptidyl peptidase IV (DPPIV) of *O. mirabilis* UM 578 (UM578_9285). Alignment was carried out with TruDPPIV from *Trichophyton rubrum* (AAS76665) and AfuDPPIV from *Aspergillus fumigatus* (AAC34310). Identical and similar residues are black and gray shaded respectively. The catalytic triad is indicated in asterisk (Ser619, Asp 696, His731).

```
UM578_9264          1 MVSFKSAATAVALALPFVRAITPEQMLAAPRRSEAIPNPSGEWAAFTSTTYSWETHEAST
AAN03632_TruDPPV    1 MAAAKWLIASLAFASSGL-AFTPEDFISAPRRGEAIPDPKGELAVFHVSKYNFDKKDRPS
AAB67282_AfuDPPV    1 MGAFRWLSIAAA-ASTAL-ALTPEQLITAPRRSEAIPDPSGKVAVFSTSQYSFETHKRTS

UM578_9264         61 VWNLMNLTSGEVSLLFNGSDISEMVWIGPTDTSVIYVNGTNAEEDGGISLYAADVTAIEN
AAN03632_TruDPPV   60 GWNLLNLKNGDINVLTTDSDVSEITWLGD-GTKVVYINGTDS-VKGGVGIWISDAKNFGN
AAB67282_AfuDPPV   59 WWSLLDLKTGQTKVLTNDSSVSEIVWLSD--DSILYVNSTNADIPGGVELWVTQASSFAK

UM578_9264        121 ATLIGSLPAPYSGMKAVQAANGD-VHFVMYCQAYVNGTAYNAELEQEPLSTARIYTDIYV
AAN03632_TruDPPV  118 AYKAGSVNGAFSGLKLAK--SGDKINFVGYGQSTTKGDLYNEAAAKEAVSSARIYDSLFV
AAB67282_AfuDPPV  117 GYKAASLPASFSGLKAAKTKSGD-IRFVAYGQSYPNGTAYNEELATAPLSSARIYDSIYV

UM578_9264        180 RHWDTWLTDKKNNVFSGVLTSSD--GGYSFDGNMTNLMAGLSNVTRAESPVMPFGGSGDY
AAN03632_TruDPPV  176 RHWDTYVGTQFNAVFSGTLTKSG--DKYSFDGKLKNLVQP---VKYAESPYPPFGGSGDY
AAB67282_AfuDPPV  176 RHWDYWLSTTFNAVFSGTLKKGHGKNGYSLDGELKNLVSP---VKNAESPYPPFGGASDY

UM578_9264        238 DISPDGNQVAFLTKNIDLTLANYTSSQIWLVPFGGGE-PQVLNGLGAASTPANAEGASAG
AAN03632_TruDPPV  231 DLSSDGKTVAFMSKAPELPKANLTTSYIFLVPHDGSRVAEPINKRNGPRTPQGIEGASSS
AAB67282_AfuDPPV  233 DLSPDGKWVAFKSKAPELPKANFTTSYIYLVPHDASETARPINGPDSPGTPKGIKGDSSS

UM578_9264        297 PVFSPDSTKIAYLQMDEIAYESDKNKIYVANADAANPEIQVLVEDWDVSPSALKWNTNGT
AAN03632_TruDPPV  291 PVFSPDGKRIAYLQMAAKNYESDRRVIHIAEVGTNK-PVQRIASNWDRSPEAVKWSSDGR
AAB67282_AfuDPPV  293 PVFSPNGDKLAYFQMRDETYESDRALLYVYSLGSKK-TIPSVAGDWDRSPDSVKWTPDGK

UM578_9264        357 GLFLDAPHRGNDRMFELIPLDAPADLEPANITDRGTTAAFFVLPDD-NLLVSDSKAWSAR
AAN03632_TruDPPV  350 TLYVTAEDHATGKLFT-LPADARDNHKPAVVKHDGSVSSFYFIGSSKSVLISGNSLWSNA
AAB67282_AfuDPPV  352 TLIVGSEDLGRTRLFS-LPANAKDDYKPKNFTDGGSVSAYYFLPDS-SLLVTGSALWTNW

UM578_9264        416 DVYIISPTGEVVADLFHANEVDPGYEGLDPSIITEFYYPGNFTDVQAFIVYPQDFDESKK
AAN03632_TruDPPV  409 LYQVATPDRPN-RKLFYANEHDPELKGLGPNDIEPLWVDGARTKIHSWIVKPTGFDKNKV
AAB67282_AfuDPPV  410 NVYTAKPEKGVIKKIASANEIDPELKGLGPSDISEFYFQGNFTDIHAWVIYPENFDKSKK

UM578_9264        476 YPLAFIIHGGPQVQHSNAWSTRWNFKVWADQGYVVVAPNPTGSNGFGEAFQDAITNNWGG
AAN03632_TruDPPV  468 YPLAFLIHGGPQGSWGDNWSTRWNPRVWADQGYVVVAPNPTGSTGFGQKLTDDITNDWGG
AAB67282_AfuDPPV  470 YPLIFFIHGGPQGNWADGWSTRWNPKAWADQGYVVVAPNPTGSTGFGQALTTAIQNNWGG

                                                    *
UM578_9264        536 YPYDDLVKCHAYVKENFPYIDTENGIAAGASYGGYMTNWIQGHDLGREFKALVTHDGSTD
AAN03632_TruDPPV  528 APYKDLVKIWEHVHDHIKYIDTDNGIAAGASFGGFMVNWIQGQDLGRKFKALVSHDGTFV
AAB67282_AfuDPPV  530 APYDDLVKCWEYVHENLDYVDTDHGVAAGASYGGFMINWIQGSPLGRKFKALVSHDGTFV

                                                                           *
UM578_9264        596 TLAQYTSEELWFMEHDFNGTLWNDRENYERWNPI--NHILNWATPQFVVHNTLDYRLPES
AAN03632_TruDPPV  588 GSSKIGTDELFFIEHDFNGTFFEARQNYDRWDCSKPELVAKWSTPQLVVHNDFDFRLSVA
AAB67282_AfuDPPV  590 ADAKVSTEELWFMQREFNGTFWDARDNYRRWDPSAPERILQFATPMLVIHSDKDYRLPVA

                                               *
UM578_9264        654 EGIMLFNILQRRGVPSKFLSFPDENHWVLNRENSLVWHTEIYKWINYYSGLSQESPF---
AAN03632_TruDPPV  648 EGVGLFNVLQEKGVPSRFLNFPDETHWVTKPENSLVWHQQVLGWVNKWSGINKSNPKSIK
AAB67282_AfuDPPV  650 EGLSLFNVLQERGVPSRFLNFPDENHWVVNPENSLVWHQQALGWINKYSGVEKSNPNAVS

UM578_9264            -------------------
AAN03632_TruDPPV  708 LSDCPIEVVDHEAHSYFDY
AAB67282_AfuDPPV  710 LEDTVVPVVNYN-------
```

Fig. S10 Alignment of predicted dipeptidyl peptidase V (DPPV) of *O.mirabilis* UM 578 (UM578_9264). Alignment was carried out with TruDPPIV from *Trichophyton rubrum* (AAN03632) and AfuDPPIV from *Aspergillus fumigatus* (AAB67282). Identical and similar residues are black and gray shaded respectively. The catalytic triad is indicated in asterisk (Ser566, Asp647, His679).

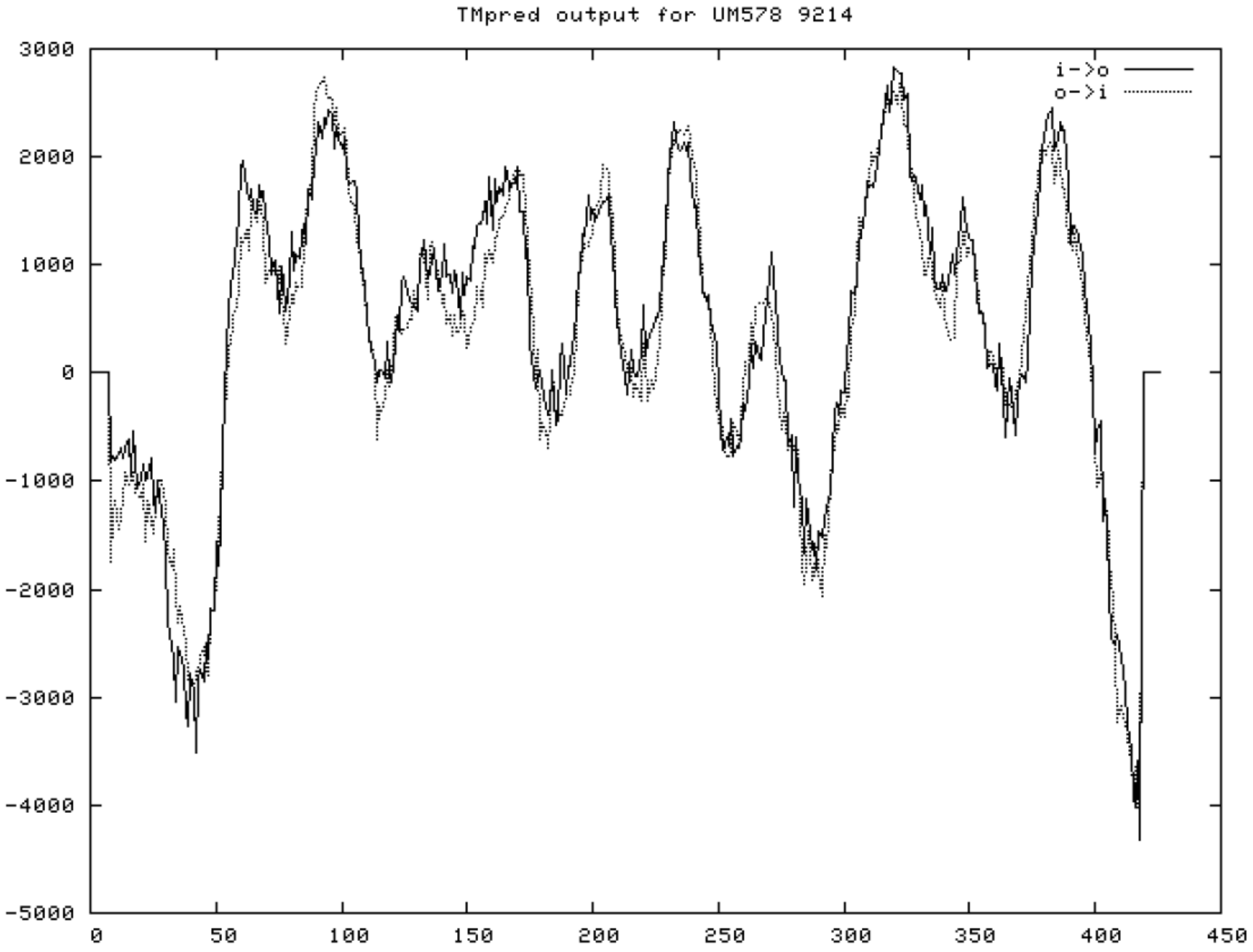

Fig. S11 TMpred output of putative sulphite efflux pump (*ssu1*) in UM 578. The putative gene, UM578_9214 has ten membrane-spanning helixes and hydrophilic N- and C- termini.

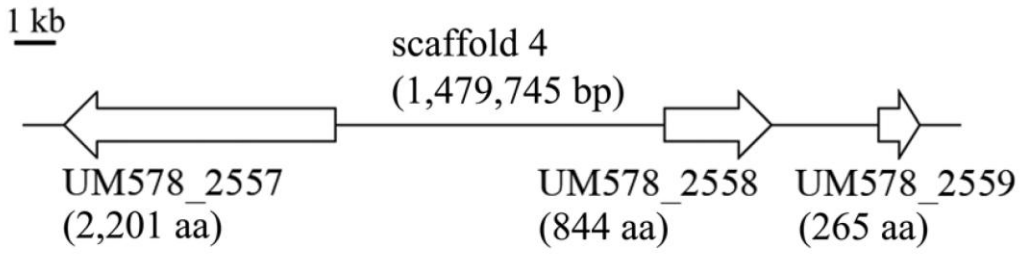

Fig. S12 Putative melanin biosynthesis cluster in UM 578. The organisation and orientation of genes involved in melanin biosynthesis are similar to that of the reported melanin gene cluster in *C. heterostrophus* [GenBank: AAR90272] and *A. brassicicola* [GenBank: BAD22832]. The predicted genes encode polyketide synthase (UM578_2557), transcription factor Cmr1 (UM578_2558) and tetrahydroxynaphthalene reductase (UM578_2559). The direction of transcription is indicated by the arrow for each gene.

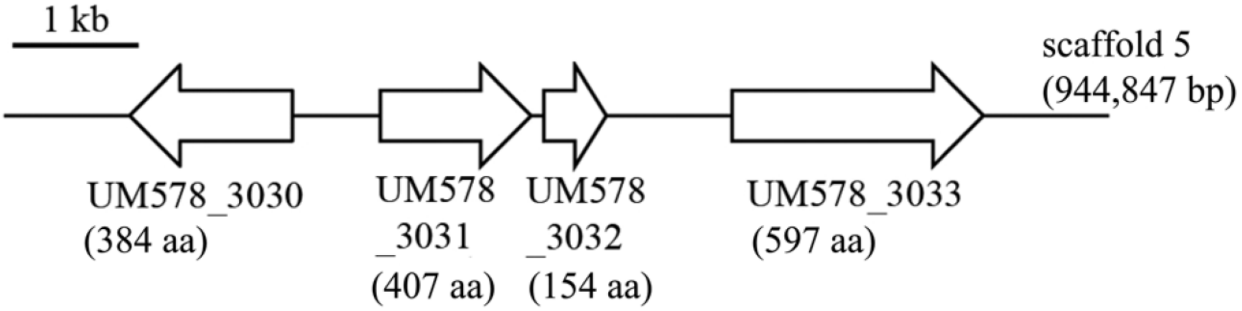

Fig. S13 Putative trichothecene biosynthesis cluster in UM 578. The 6751 bp cluster encompasses the trichodiene synthase (UM578_3030) with two cytochrome P450 encoding genes (UM578_3031 and UM578_3032) and the trichothecene efflux pump (UM578_3033). The direction of transcription is indicated by the arrow for each gene.

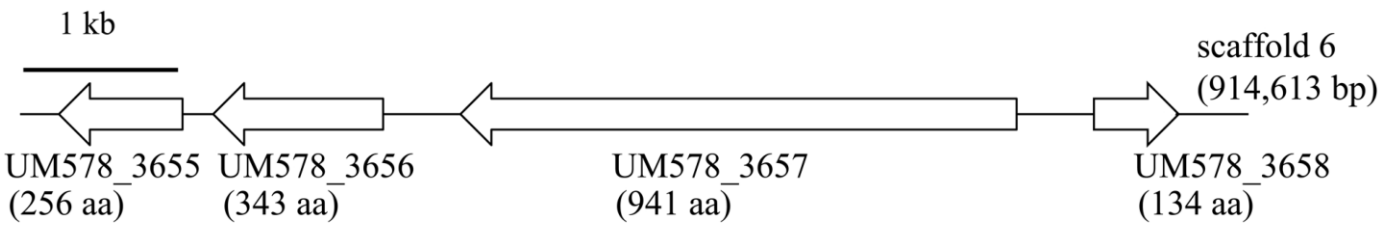

Fig. S14 Putative gene organisation of mating type genes in UM 578. The neighbouring genes of alpha-domain containing gene (UM578_3656) encompass the homeodomain-containing protein (UM578_3655), DNA lyase APN2 (UM578_3657) and cytochrome C oxidase Vla Cox13 (UM578_3658). The direction of transcription is indicated by the arrow for each gene.

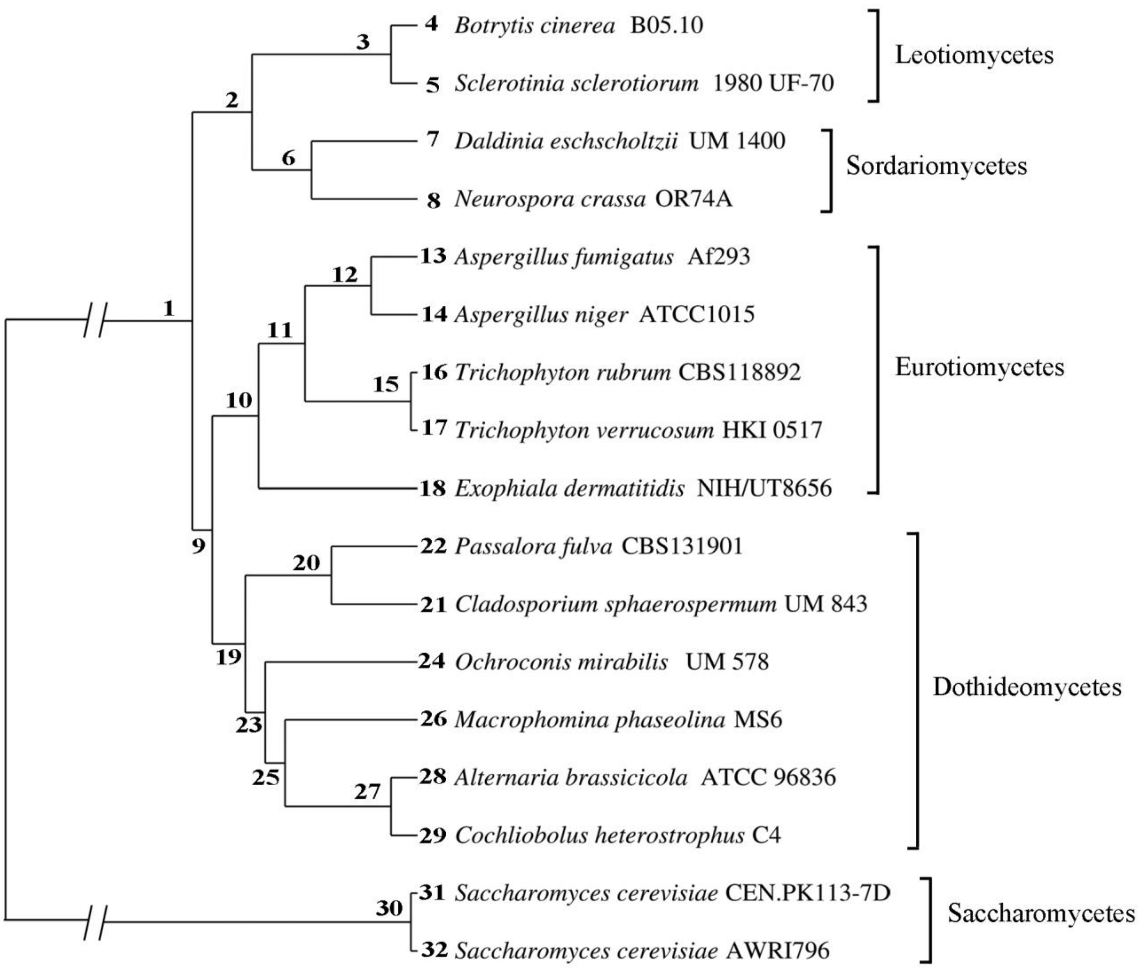

Fig. S15 Phylogenomic tree showing number of each node in expansion/ contraction analysis. The number of genes and P-value for UM 578 (node 24) and the internode (node 23) are shown in Table S12.
